# Tissue origins of the plasma proteomic response to glucose ingestion in humans

**DOI:** 10.1007/s00125-026-06800-8

**Published:** 2026-07-17

**Authors:** Burulça Uluvar, Alice Williamson, Kristoffer J. Kolnes, Per Bendix Jeppesen, Anders J. Kolnes, Mine Koprulu, Martijn Zoodsma, Carl Beuchel, Yanki Bambal, Mar Reines, Summaira Yasmeen, Carlo Maj, Johannes Schumacher, Stephen O’Rahilly, David A. van Heel, Sina Bartfeld, Julia Carrasco-Zanini, Jørgen Jensen, Maik Pietzner, Claudia Langenberg

**Affiliations:** 1 Computational Medicine, Berlin Institute of Health at Charité – Universitätsmedizin Berlin, Berlin, Germany; 2 Precision Healthcare University Research Institute, Queen Mary University of London, London, UK; 3 Friede Springer Cardiovascular Prevention Center at Charité, Charité University Medicine Berlin, Berlin, Germany; 4 Steno Diabetes Center, Odense University Hospital, Odense, Denmark; 5 Department of Physical Performance, Norwegian School of Sport Sciences, Oslo, Norway; 6 Department of Clinical Medicine, Aarhus University, Aarhus, Denmark; 7 Faculty of Medicine, University of Oslo, Oslo, Norway; 8 Department Medical Biotechnology, Institute of Biotechnology, Technische Universität Berlin, Berlin, Germany; 9 Center for Human Genetics, University Hospital of Marburg, Marburg, Germany; 10 MRC Metabolic Diseases Unit, Institute of Metabolic Science, University of Cambridge, Cambridge, UK; 11 Blizard Institute, Queen Mary University of London, London, UK; 12 Max Planck Institute for Molecular Genetics, Berlin, Germany

**Keywords:** Fasting, Gastric acid, Glucose tolerance, Gut hormones, Intervention study, Multi-omics, Population studies, Proteomics

## Abstract

**Aims/hypothesis:**

Circulating proteins act as important hormonal signals of nutrient intake. We aimed to systematically characterise the time-resolved proteomic response to glucose ingestion in humans, and to assess its robustness following prolonged complete caloric restriction.

**Methods:**

We conducted oral glucose tolerance tests (OGTTs) in 11 healthy volunteers before and after 7 days of complete caloric restriction and measured the response of >2900 targets through high-resolution plasma protein profiling.

**Results:**

We identified a signature of 44 proteins that changed significantly following glucose ingestion, which was reproducible after 7 days without food, and was strongly (20-fold) enriched for ‘stomach-specific’ proteins. We report that annexin A10 (ANXA10) shows the most significant post-glucose change observed, similar to the trajectories of secreted hormones. We present observational human evidence from multiple sources suggesting that ANXA10 is secreted upon sensing an increase in gastric pH, with the stomach as the major contributing tissue. Despite a profound metabolic shift after 7 days of complete caloric restriction, characterised by delayed insulin secretion and postprandial hyperglycaemia, only four proteins showed robust evidence for a differential trajectory during both OGTTs. This included plasma levels of tryptophanyl-tRNA synthetase 1 (WARS), for which we found a genetic association with glucose homeostasis and coronary artery disease.

**Conclusions/interpretation:**

Our exploratory study identifies the proteomic response to glucose ingestion and demonstrates its reproducibility despite major shifts in glucose homeostasis. We characterise the gastrointestinal origin of these changes, and hypothesise a hitherto under-recognised role for sensing of changes in gastric pH on the plasma proteome.

**Graphical Abstract:**

**Figure:**
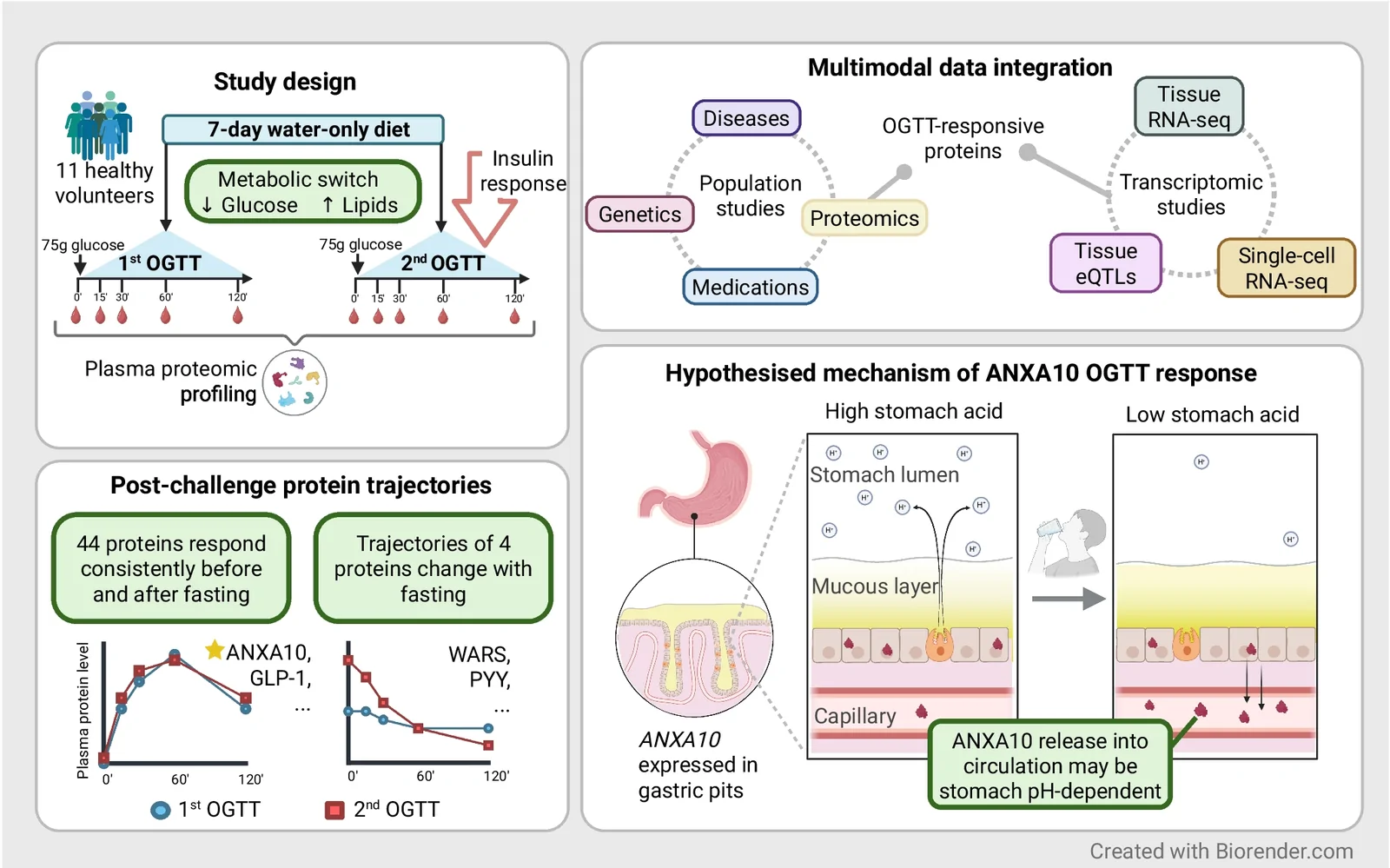

**Supplementary Information:**

The online version contains peer-reviewed but unedited supplementary material available at https://doi.org/10.1007/s00125-026-06800-8.

**Figure:**
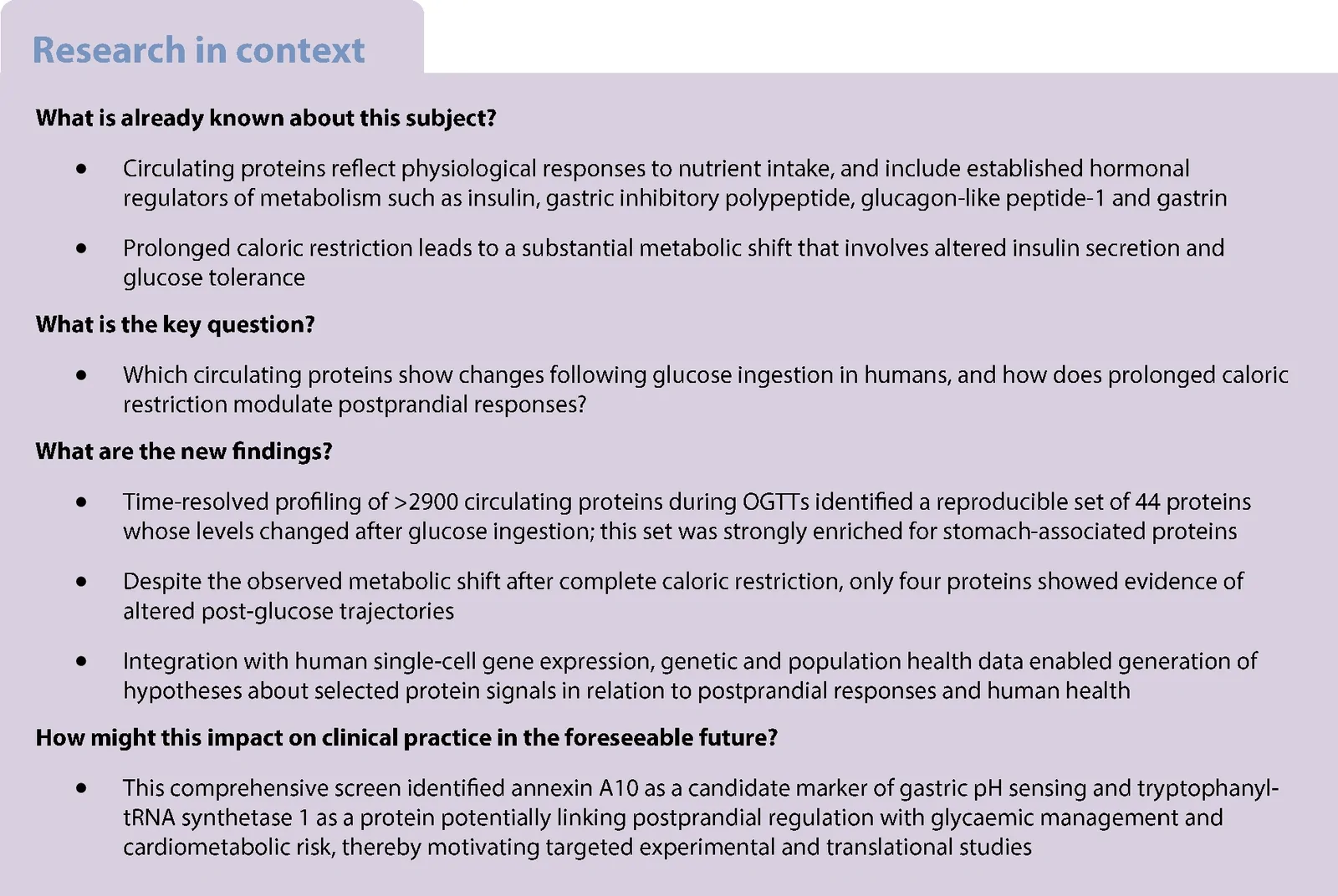

## Introduction

The switch between the fasted and fed state is at the core of human metabolism, yet our understanding of how the body senses and responds to nutrient intake at the molecular level remains incomplete. Postprandial processes are initiated mainly by the gastrointestinal tract, and gut-derived hormones (incretins) signal nutrient availability, initiating digestive and metabolic processes, such as gastric emptying. These incretins are further crucial for decreasing postprandial glucose levels by enhancing pancreatic beta cell insulin release. Drugs mimicking incretin signalling, such as the glucagon-like peptide-1 (GLP-1) receptor agonist semaglutide, improve insulin secretion in type 2 diabetes patients [1]. These drugs have transformed the pharmacological treatment of obesity, demonstrating the potential of better understanding these circuits for human health and disease [2].

The evolution of human metabolism was driven by days to weeks of little or no food, prompting a transition in fuel utilisation from external glucose intake to mobilisation of lipids from internal storage. This transition is characterised by physiological changes in glucose tolerance upon refeeding that can mimic postprandial hyperglycaemia [3], probably due to insulin resistance in skeletal muscle [4], enhanced hepatic insulin sensitivity, and reduced pancreatic insulin secretion [5]. These physiological changes generate a unique opportunity to study important mediators of metabolic flexibility to improve our understanding of human metabolism, and subsequently guide the development of targeted strategies for metabolic diseases. We have recently demonstrated profound time-dependent changes during complete caloric restriction for plasma levels of >1000 proteins involved in multiple endocrine axes but also other body systems, with major systemic changes becoming evident only after several days [6], and provided evidence that upregulation of pyruvate dehydrogenase kinase 4 (PDK4) may represent a counter mechanism in muscle to avoid hypoglycaemia [7].

Here, using samples obtained during the same intervention study, we systematically characterised the plasma protein response to a standardised oral glucose tolerance test (OGTT) before and after 7 days of complete caloric restriction. We integrated data from population-scale and tissue-/single cell-level transcriptomic studies to describe the tissue origins and modulators of the plasma levels of proteins that change upon glucose intake.

## Methods

For detailed methods, please refer to the electronic supplementary material (ESM) Methods.

### Study design

**Fig. 1:**
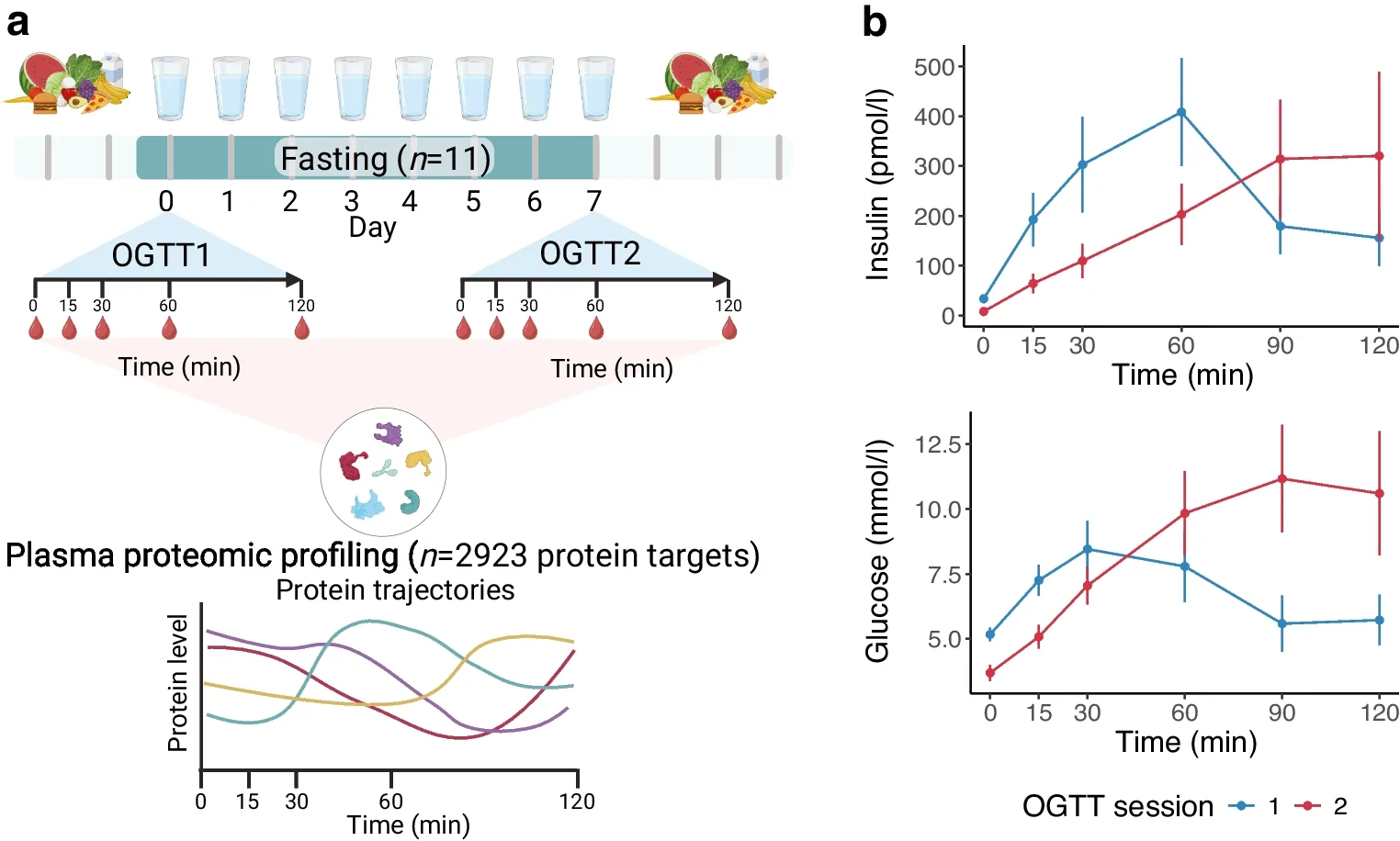
Study design and glucose response. (**a**) Study design. Eleven participants fasted for 7 days and underwent two OGTTs: OGTT1 on day 0 and OGTT2 on day 7. Plasma samples were collected at five timepoints during each OGTT for proteomic analysis. Created with BioRender.com. (**b**) Dynamic changes in plasma insulin and glucose concentrations during both OGTT sessions. Error bars indicate 95% CIs. Linear mixed model testing for whether trajectories differed between OGTT sessions resulted in a *p* value for insulin of 4.8 × 10^−11^ and a* p* value for glucose of 2.0 × 10^−17^

We performed OGTTs (75 g glucose dissolved in 300 ml water) on 11 apparently healthy, normoglycaemic, non-smoking participants of self-reported European descent at two timepoints: after an overnight fast, and after a 7 day diet that allowed only ad libitum water intake (Fig. 1a). We refer to these subsequently as ‘OGTT1’ and ‘OGTT2’, respectively. The exclusion criteria were any known disease and percentage body fat below 15% for female participants and 12% for male participants. To examine potential sex-differential effects, we included five women and six men for whom self-reported sex agreed with sex-specific proteomic profiles. Participant characteristics are shown in ESM Table 1. We have already demonstrated good adherence of participants to the fasting scheme in the absence of adverse events [6], but note that one female participant did not complete OGTT2. During both OGTTs, we sampled blood from each participant at the start as well as after 15, 30, 60 and 120 min following glucose ingestion for time-resolved proteomic profiling. Plasma insulin and glucose were measured as described elsewhere [8].

All participants gave their written informed consent, and the study was approved by the local ethics committee (Norwegian School of Sport Sciences, no. 15-220817).

### Proteomics measurements

Using the Olink 3072 Explore platform, we measured the plasma levels of 2923 unique protein targets across 2941 assays in 105 samples, and applied standard quality control. Details are provided in ESM Methods.

### Statistical analysis of glucose, insulin and protein trajectories

We tested for time-varying effects during each OGTT, differential effects across OGTTs, and sex-differential effects on insulin, glucose and protein levels before and after fasting using linear mixed models. We used *z* score-standardised NPX values for each protein using the mean and SD at minute 0 of OGTT1, such that values represent SD units relative to OGTT1 minute 0. We used a false discovery rate (*q* value) threshold of 5% across all proteins, except for the interaction analysis where we used 20% to assign statistical significance. Details are provided in ESM Methods.

### Insulin indices

We calculated 17 surrogate measures of insulin sensitivity and beta cell function using the glucose or insulin measurements taken each day during fasting or during the two OGTTs. Details are provided in ESM Methods.

### Tissue enrichment analysis

To explore the potential tissue origins of the OGTT-responsive proteins, we performed Fisher’s exact tests using tissue specificity data from the Human Protein Atlas (https://www.proteinatlas.org/, accessed on 24 July 2023). Details are provided in ESM Methods.

### Participant characteristics and *trans*-pQTL enrichment analyses

We used the ‘enrichment_test_across_groups()’ function from the ‘prodente’ (version 0.2.0) package (https://github.com/comp-med/r-prodente) to evaluate enrichment of protein signatures associated with various aspects of human health and disease [9]. To explore regulatory origins, OGTT signatures were tested for over-representation of proteins linked to one or more of 652 pleiotropic protein quantitative trait loci (pQTL) [10]. Details are provided in ESM Methods.

### Genetic and transcriptomic analyses

To dissect the genetic regulation, tissue and cellular origins, and disease associations of candidate proteins identified in the analysis of the OGTT response, we (1) performed a genome-wide association study of annexin A10 (ANXA10) plasma levels in UK Biobank; (2) tested for a shared genetic association of plasma protein levels with gene expression in tissues [11, 12], disease risk [11, 13, 14] and biomarker levels [15]; and (3) obtained single-cell expression profiles from gastrointestinal tissues [16] and pancreatic tissues [17]. Details are provided in ESM Methods.

### Phenotypic associations with plasma protein levels in UK Biobank

We evaluated the influence of prevalent gastric diseases and intake of acid-suppressing drugs on protein levels in 52,140 UK Biobank participants with Olink data, using linear models adjusted for age and sex [18]. Details are provided in ESM Methods.

### Protein association profile of gastrointestinal health SNPs

To systematically profile the proteomic impact of SNPs linked to gastrointestinal health, we extracted summary statistics from the UK Biobank Pharma Proteomics Project [10] for independent SNPs that were associated with gastric phenotypes and were *cis*- or *trans*-pQTLs for at least one protein, based on our previous work [9]. Details are provided in ESM Methods.

### ANXA10 pLOF carrier in the Genes & Health cohort

In the Genes & Health cohort of 44,028 exome-sequenced British Pakistani and Bangladeshi participants [19], we identified a single individual homozygous for a predicted loss-of-function (pLOF) variant in ANXA10 (rs752829627), with clinical diagnoses verified through linked electronic health records. Details are provided in ESM Methods.

## Results

Participants were normoglycaemic and insulin-sensitive at the start of the study (fasting glucose 5.2 ± 0.4 mmol/l; mean fasting insulin 33.7 ± 9.5 pmol/l; 2 h glucose 5.7 ± 1.4 mmol/l; means ± SD; Fig. 1b). During OGTT2, participants showed a delayed and blunted insulin response and signs of postprandial hyperglycaemia (2 h glucose 10.6 ± 3.3 mmol/l) (ESM Table 2), in line with previous observations of day-long fasting [3, 5]. Refined measures pointed towards increased muscle insulin resistance but increased hepatic insulin sensitivity (ESM Fig. 1, ESM Table 3). We did not observe sex-differential effects on glucose or insulin levels during either OGTT session (*p*>0.05).

### Trajectories during OGTTs reveal a conserved proteomic response

**Fig. 2:**
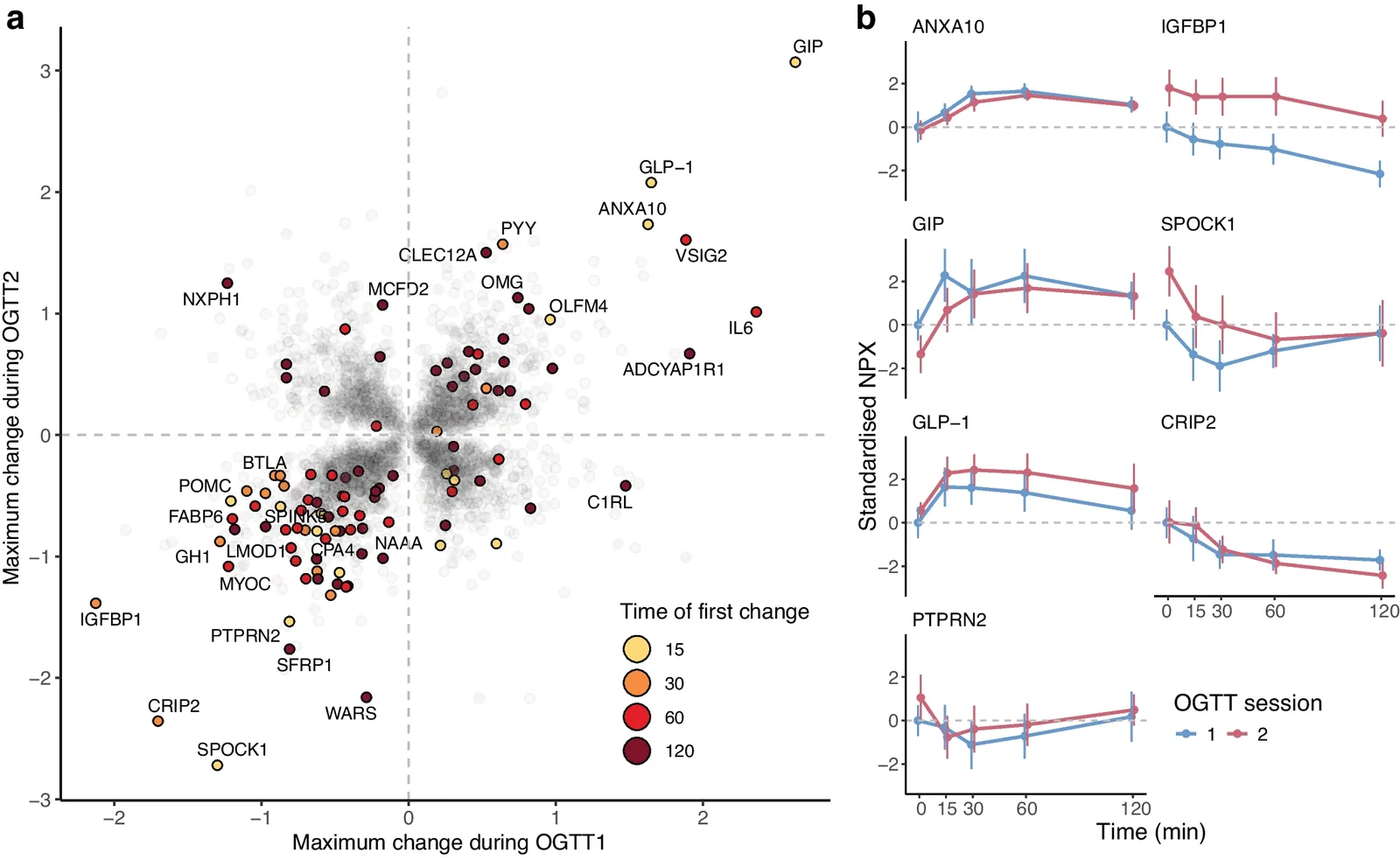
Plasma proteome response to an OGTT before and after a 7 day fast. (**a**) Highest change (in SD units) for each protein target in OGTT1 vs OGTT2. Points are coloured according to the time point at which the protein first changed significantly during either OGTT. (**b**) Plasma trajectories of proteins that responded within the first 30 min and show a change greater than 1.5 SD units. Error bars indicate 95% CIs

We observed 52 and 71 proteins (106 in total) for OGTT1 and OGTT2, respectively, that showed significantly altered plasma levels (*q* value <5%) at one or more timepoints during the OGTT (Fig. 2a, ESM Fig. 2a, ESM Table 4). Despite the profound changes in metabolism induced by 7 days of fasting, a total of 44 proteins showed evidence of a conserved response across both OGTTs, and four proteins showed robust evidence of a differential response (see ‘Differential proteomic response during an OGTT after a 7 day fast’ section below). We did not observe sex-differential effects on protein levels for either OGTT.

We detected eight proteins with large changes (|maximum change| >1.5 SD) within the first 30 min, possibly indicating active secretion or depletion, while most proteins showed small and late changes (Fig. 2b, ESM Fig. 2b). Among the early responders were regulators of glucose homeostasis and insulin response, namely gastric inhibitory polypeptide (GIP), GLP-1 and insulin like growth factor binding protein 1 (IGFBP1) [20]. We identified an as yet unreported protein in response to an OGTT, ANXA10 (peak of +2.77 SD at 60 min), which showed the statistically most significant postprandial change, with a remarkably conserved trajectory across both experiments and individuals (ESM Fig. 2c). Other so far unreported findings comprised a decrease in plasma levels of testican-1 (SPOCK1; maximum change of −1.66 SD at 60 min), phogrin (PTPRN2; maximum change of −1.02 SD at 15 min) and cysteine-rich protein 2 (CRIP2; maximum change of −1.70 SD at 120 min).

We further tested whether changes in the proteome associated with changes in plasma glucose and insulin levels, but did not find evidence for consistent associations across both OGTTs (ESM Table 5). These results suggest that the observed response in the proteome is probably independent of insulin secretion or glucose uptake.

### Gastrointestinal tissues contribute to the proteomic changes during an OGTT

We next contextualised the protein responses during the OGTT by testing them for enrichment among tissue and regulatory signatures. First, we observed an approximately 20-fold enrichment of protein targets with enriched or enhanced expression of corresponding mRNA levels in stomach in both OGTT sessions (OGTT1: OR 20.6, *q* value=8.7 × 10^−5^; OGTT2: OR 18.2, *q* value=3.2 × 10^−5^) (ESM Fig. 3a, ESM Table 6). Among those were important regulators of appetite and digestive function such as ghrelin and gastrin, but also ANXA10 (ESM Fig. 3b). Intestine-specific proteins were significantly enriched only in OGTT2 (OR 4.0, *q* value=0.03).

Second, we observed a >20-fold enrichment of proteins associated with the intake of various proton pump inhibitors (PPIs; e.g. OR 23.0, *q* value=1.5 × 10^−6^ for omeprazole) among proteomic signatures linked to various aspects of human health and disease [9]. (ESM Fig. 4a, ESM Table 7). The participants did not take any drugs during the course of the study. We also observed an enrichment of proteins associated with random glucose measurements (OR 5.9, *q* value=1.4 × 10^−4^) that was mostly distinct from the PPI-associated signature.

Third, pQTLs have been shown to link plasma protein abundance to tissue processes [10], and we observed that five independent pQTLs had significantly enriched protein signatures (*q* value < 5%) among various protein sets changing during the OGTT. These responses included >30-fold enrichment among proteins with a conserved response during the OGTT, including genes such as *PSCA* (e.g. rs2920282: OR 71.5, *q* value=1.9 × 10^−3^), *LILRB5* (e.g. rs12986064: OR 33.4, *q* value=1.8 × 10^−3^) or *CYP7A1* (e.g. rs4738679: OR 40.9; *q* value=6.5 × 10^−3^) (ESM Table 8). Rapid downregulation of *CYP7A1* during an OGTT has been reported previously [21]. *PSCA* encodes a stomach-enriched protein [22], and the same genetic variant is associated with susceptibility to peptic ulcers in various ancestral groups [23, 24].

### Human genetic and single-cell evidence identify ANXA10 at the core of an OGTT-associated protein set common to pancreas and stomach

To identify possible tissue origins of the most significant but unreported plasma protein ANXA10, we performed genome-wide association testing of plasma levels of ANXA10 in 46,929 individuals in UK Biobank. We observed two distinct loci that reached genome-wide significance (*p*<5.0 × 10^−8^) (ESM Fig. 5, ESM Table 9). These suggested partly distinct tissues of origin once we integrated information on genetically regulated tissue mRNA expression [11, 12].

Briefly, a common intronic variant in *ANXA10* (rs4600882) showed evidence of increasing plasma protein levels (β=0.04, *p*=2.14 × 10^−10^) and corresponding gene expression in the liver, oesophagus muscularis, pancreas and gastro-oesophageal junction but not the stomach (ESM Fig. 6a, ESM Table 10), which is the tissue with the highest *ANXA10* mRNA expression (ESM Fig. 7a). In further support of a non-stomach origin of plasma ANXA10, we observed lower, but still reliably detectable, plasma ANXA10 levels in UK Biobank participants who underwent gastrectomy before blood samples were taken (ESM Fig. 7b). However, a contribution of the stomach to circulating plasma ANXA10 levels was supported by the second independent variant (rs2990223) acting in *trans*, probably via *MUC1*, which encodes mucin 1 [11, 25]. (ESM Fig. 6b; ESM Table 11).

**Fig. 3:**
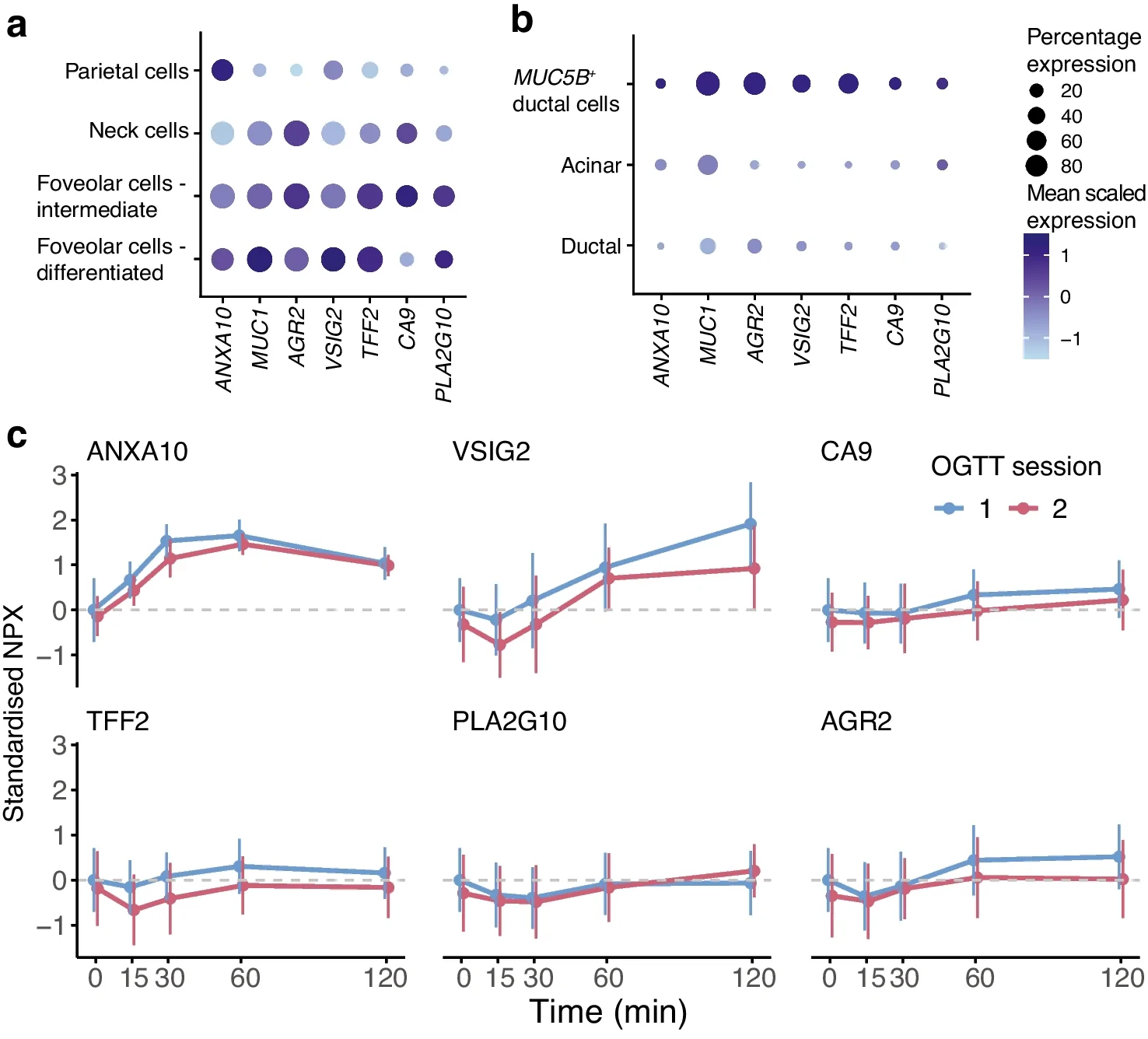
Cell type origins of plasma ANXA10. Gene expression dot plots for *ANXA10* and a set of co-expressed genes in relevant cell types from public single-cell RNA-seq datasets: (**a**) gastrointestinal single-cell data from Nowicki-Osuch et al [16]. (**b**) Pancreas single-cell data from Elgamal et al [17]. (**c**) Trajectories of ANXA10 and co-expressed proteins whose plasma levels changed during the OGTT. Error bars indicate 95% CIs

We next refined the specific expression of *ANXA10* based on publicly available scRNA-seq data. In the gastrointestinal tract, expression was localised to mucus- and acid-secreting foveolar and parietal cells of the gastric pits (Fig. 3a, ESM Fig. 8a). In the pancreas, we observed restricted *ANXA10* mRNA expression in a subcluster of *MUC5B*-positive ductal cells (1% of ductal cells enriched for mucus secretion genes [26]) (Fig. 3b, ESM Fig. 8b). Both cell types shared a co-expression signature that included putative interaction partners [27] of *ANXA10*, such as *MUC5AC*, *AGR2*, *TFF2*, *CTSE* and *VSIG1* (ESM Fig. 9, ESM Tables 12 and 13), possibly indicating a shared function of these stomach and exocrine pancreatic cells.

The protein products of five genes co-expressed with *ANXA10* changed significantly during both OGTTs (Fig. 3c), including three that were also associated with PPI intake in UK Biobank, as described in the previous section (ESM Table 7). However, the plasma trajectories of these proteins during the OGTTs did not correlate with the change in ANXA10, arguing against coordinated release into the circulation, although functional links remain plausible. Carbonic anhydrase 9 (CA9), for example, facilitates gastric acid secretion by catalysing CO_2_ hydration, thereby increasing proton availability at the basolateral membrane of parietal cells [28]. Collectively, these findings suggest a link between gastric pH regulation and ANXA10 in the relevant cell type.

### Phenotypic correlations with ANXA10 plasma levels point to stomach acidity and mucosal integrity as potential regulators

**Fig. 4:**
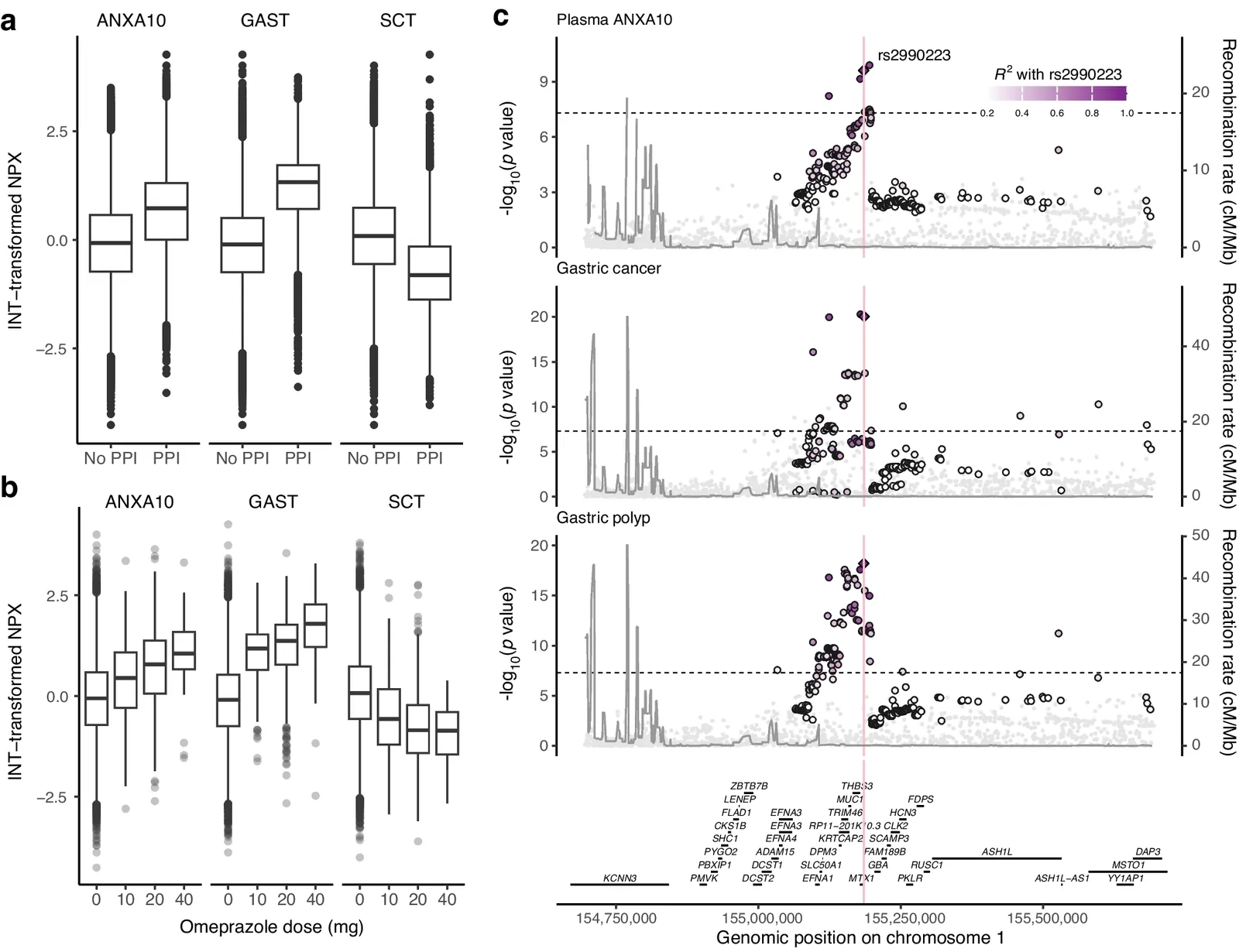
Phenotypic correlations with ANXA10 plasma levels. (**a**) Plasma levels of ANXA10 and pH-sensitive hormones in UK Biobank participants who reported taking PPI or not. (**b**) Plasma levels of the same proteins by omeprazole dose. Boxes show the median and IQR, and the whiskers extend to the furthest point within 1.5×IQR. (**c**) Regional association plot of 1 Mb region around the *trans*-pQTL (rs2990223). Gastric cancer summary statistics are from Hess et al [11] (middle); gastric polyp statistics are from Sakaue et al [14] (bottom)

We next sought to understand the potential determinants of plasma ANXA10 levels outside the context of an OGTT. Systematically considering more than 1700 participant characteristics, we identified 28 that explained approximately 8.6% of the total variance in plasma ANXA10 levels among >40,000 UK Biobank participants. We found no evidence for contributions of measures of glucose metabolism or type 2 diabetes (ESM Fig. 10). In contrast, intake of various PPIs accounted for most of the explained variance [9], and was associated with a 0.64 SD increase in ANXA10 plasma levels (*p* value<10^−300^), an effect that was by far stronger than any other tested participant characteristic (Fig. 4a). We further observed a dose-dependent relationship of chronic PPI use with ANXA10 levels in a subset of 21,563 participants with available prescription data (Fig. 4b). The dose-dependent PPI effect on circulating ANXA10 (β=0.23 for 10 mg of omeprazole, *p*=0.0001) was similar to that of gastrin (β=0.20, *p*=0.002), which is an established endocrine signal of change in gastric pH [29].

**Fig. 5:**
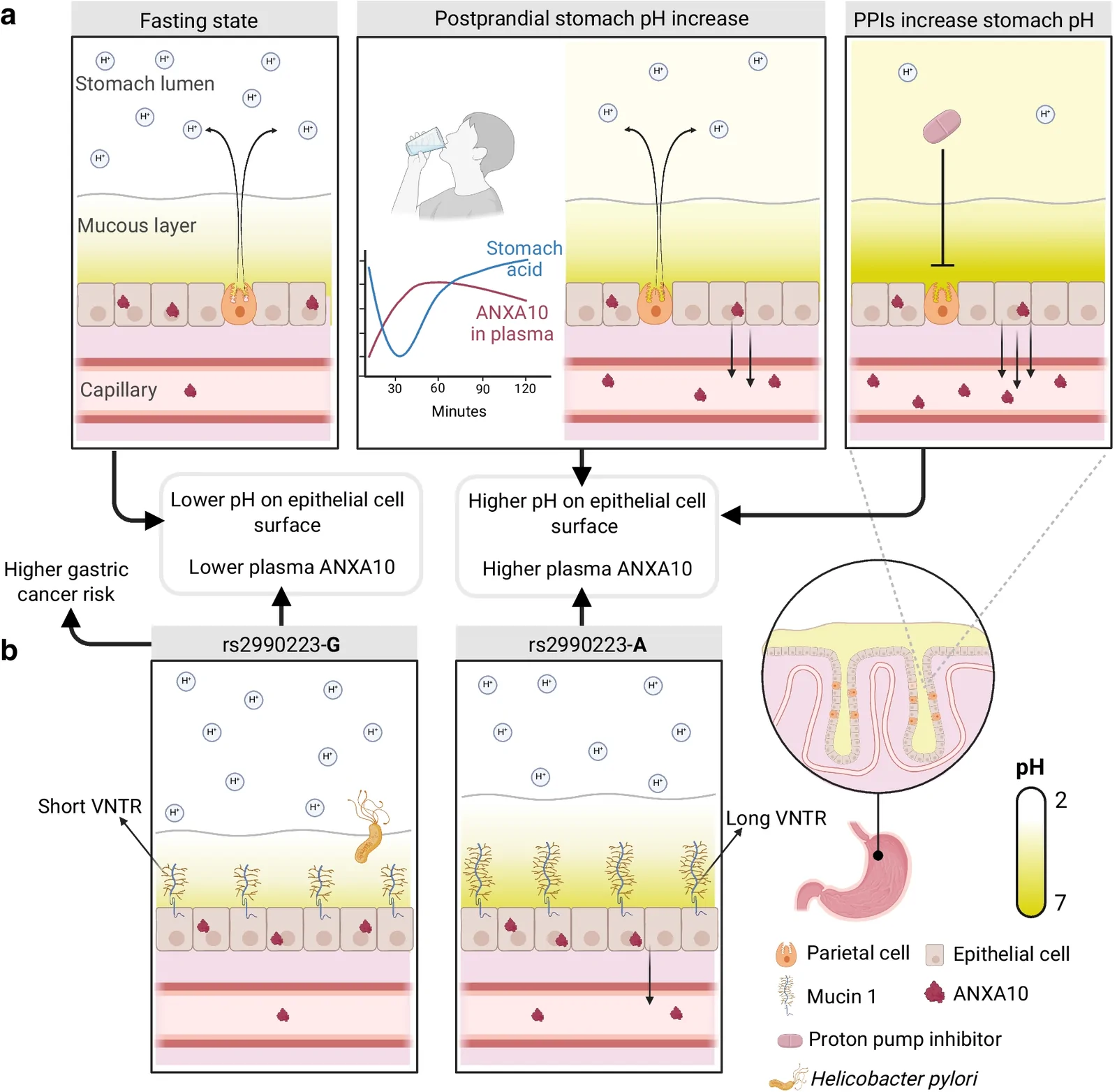
Hypothesised mechanism of ANXA10 release into the blood from the stomach. (**a**) The pH of the stomach lumen increases postprandially or after taking PPIs, which block acid release from parietal cells, compared with the fasting state. This shifts the pH gradient of the protective mucous layer towards less acidic, and ANXA10 may be released into the bloodstream in response to the epithelial cells sensing lower acidity. (**b**) The A allele of the rs2990223 variant is associated with a version of mucin 1 with a longer glycosylated extracellular domain. This may improve its barrier function against acid, in addition to its role in protection against pathogens such as *H. pylori* and susceptibility to gastric cancer. Therefore, the epithelial cells may persistently sense a higher pH and secrete higher levels of ANXA10 into the blood in people with the A allele. Created with BioRender.com

A PPI-mediated increase in gastric pH provides a potential explanation for the OGTT response of ANXA10, as the glucose solution dilutes gastric acidity [30] (Fig. 5a). This is corroborated by concordant changes in plasma levels of secretin, a pH-sensitive hormone that is secreted in the duodenum [31], during the OGTT and also in UK Biobank participants reporting PPI intake (Fig. 4b and ESM Fig. 2d). An effect of gastric pH modulation rather than stomach diseases, e.g. via tissue leakage, was further apparent when contrasting plasma ANXA10 levels among people with various gastric diseases who did not report PPI intake (ESM Fig. 4b). Notably, participants who were seropositive for *Helicobacter pylori* had elevated levels of ANXA10 even independently of PPI intake (β=0.47, *p*=1.9 × 10^−14^), possibly due to an increase in pH at infected sites of the stomach [32] (ESM Fig. 4c). We note that the *trans*-pQTL in the *MUC1*locus further provided indirect support against tissue leakage being a major contributor to plasma ANXA10 levels. Briefly, if ANXA10 were elevated in plasma simply due to leakage from damaged tissue, we would expect the disease risk-increasing allele to be associated with higher ANXA10. However, the ANXA10-increasing rs2990223 A allele co-localises with a lower risk for severe gastric diseases, most prominently gastric cancer [11] but also gastric polyps [14], mediated by a thicker mucus lining (Figs. 4c, 5b, ESM Table 14).

Finally, we identified a human homozygous pLOF carrier in *ANXA10* from the Genes & Health cohort [19]. The participant had a history of gastrointestinal disease, including duodenal ulcers, reported in their 30s, and early onset of coronary artery disease and myocardial infarction in their 40s. The homozygous pLOF variant (chr4:168164289:G>A, rs752829627) is extremely rare in Europeans (minor allele frequency=0.00017%) and most frequent in people of South East Asian ancestry (minor allele frequency=0.029%), with no homozygous carrier reported in gnomAD [33]. It is a splice donor variant with high predicted impact (CADD score 34.0), possibly disrupting splicing due to loss of a splice donor site at the 5′ of intron 5 (SpliceAI score 0.99) and activation of a cryptic splice site 17 bp downstream of the canonical splice site (SpliceAI score 0.84). Either possibility may result in an unstable or dysfunctional *ANXA10* transcript or protein.

### Differential proteomic response during an OGTT after a 7 day fast

**Fig. 6:**
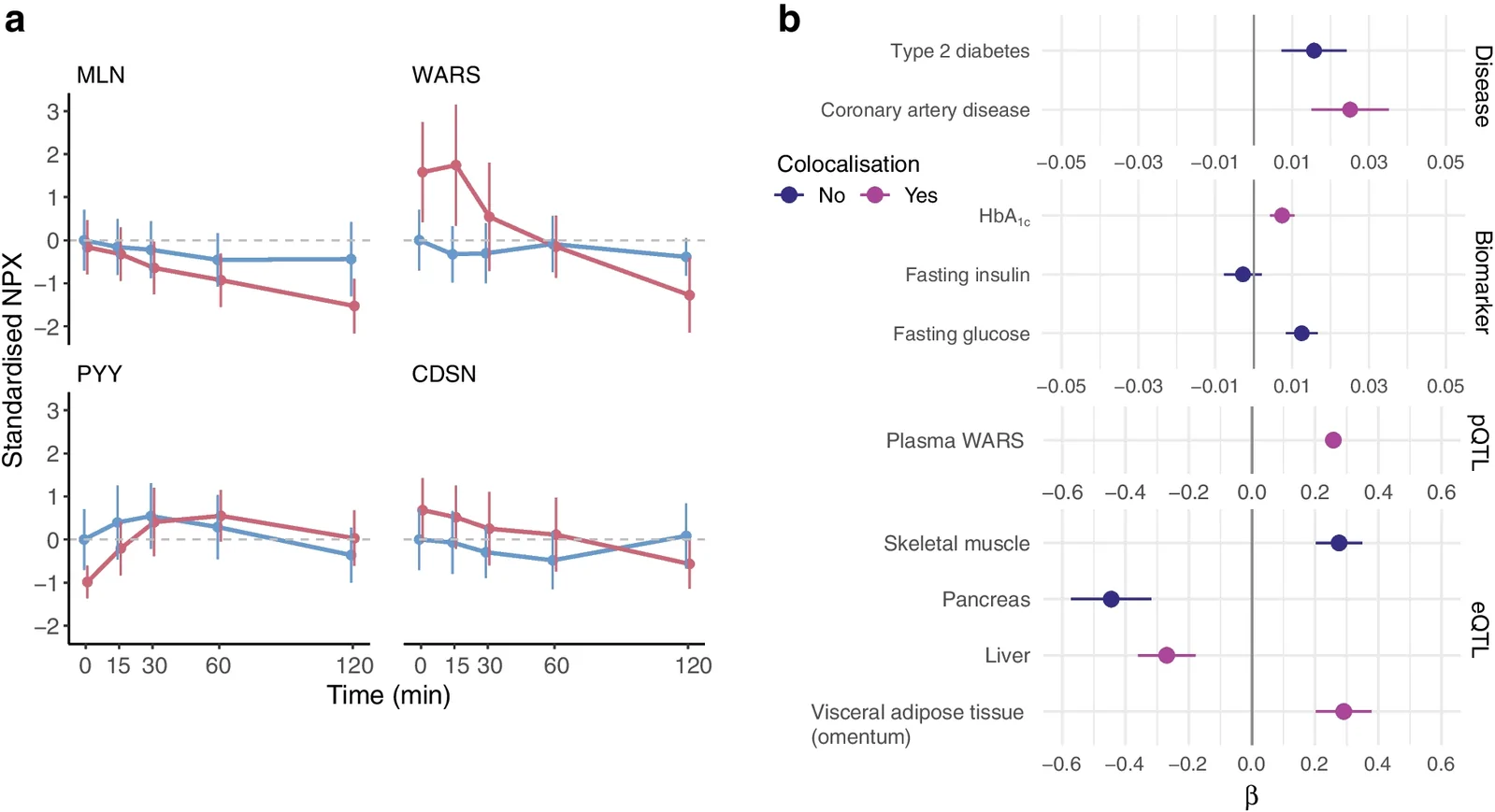
Proteins with differential trajectories for both OGTTs. (**a**) Four proteins responded differentially during the OGTT before and after the fasting intervention. Error bars indicate 95% CIs. (**b**) Forest plots of the effect sizes of the WARS pQTL (rs2273804) on tissue mRNA expression of WARS1, plasma WARS levels, blood biomarkers and disease outcomes. Note the differing *x* axis scales

We observed four proteins with robust evidence for a differential response across both OGTTs (*q* value < 20%) (Fig. 6a, ESM Fig. 2e, ESM Table 15). For three of these, the differential response may be explained by an effect of prolonged caloric restriction on their pre-OGTT plasma levels. The plasma levels of tryptophanyl-tRNA synthetase 1 (WARS) (*q*_interaction_=0.086) and corneodesmosin (CDSN, *q*_interaction_=0.13) increased, while the levels of the satiety hormone peptide YY (*q*_interaction_=0.037) decreased following the 7 day fast [34]. Pre-OGTT plasma levels of the fourth differentially responding protein, motilin (MLN, *q*_interaction_=0.05), were unaffected by prolonged caloric restriction but showed a stronger decrease in OGTT2 [35].

The differential response of WARS is noteworthy, as a common genetic variation predisposing to higher WARS levels also predisposes to higher fasting glucose and HbA_1c_ levels [15, 36, 37]. We corroborated this finding by systematically testing variants near corresponding protein coding genes for all proteins with evidence of altered plasma trajectories during either OGTT (Fig. 6b, ESM Table 16). Integrating gene expression evidence provided a tentative clue for the observed differential response due to differential effects in metabolically active tissues that are differentially responsive to insulin signalling following prolonged fasting. Briefly, the WARS-increasing rs2273804 C allele (β=0.26, *p*=1.9 × 10^−199^) was associated with higher *WARS1* mRNA expression in skeletal muscle (β=0.28; *p*=7.5 × 10^−13^) and visceral adipose tissue (β=0.29; *p*=3.5 × 10^−10^), but decreased expression in liver (β=−0.27; *p*=3.1 × 10^−8^) and pancreas (β=−0.45; *p*=6.4 × 10^−11^) (ESM Fig. 11, ESM Table 17). We further observed a previously unreported association between the rs2273804 C allele and a higher risk of coronary artery disease (PP for shared signal=97%; β=0.025, *p*=8.1 × 10^−7^). In addition to WARS, we found similar genetic evidence linking four proteins to cardiovascular disease risk that also changed during the OGTT. These included well-known mediators of atherosclerotic risk such as LPL or APOE, but only WARS also showed genetic evidence for associations with parameters of glucose homeostasis (ESM Table 16). A genetically inferred effect of plasma WARS levels on coronary artery disease risk may therefore be mediated via poor glycaemic management and associated sequelae.

## Discussion

Our understanding of how nutrient sensing regulates whole-body metabolism via peptide hormones or proteins remains incomplete despite major discoveries that have led to the development of transformative medicines. We describe here a time-resolved proteomic response to an OGTT that persisted even upon substantial rearrangement of human metabolism during 7 days of a water-only fast. We demonstrate the plausibility of our results by replicating well-known hormonal responses, and provide conceptual advances by (1) providing multiple lines of evidence in humans that gastric pH sensing is the putative mechanism driving the plasma elevation of most consistently responding proteins; (2) identifying strong early but transient responses of proteins such as phogrin, with a so far poorly understood role in acute glucose homeostasis, and (3) identifying protein candidates, such as WARS, that may contribute to an altered glucose response following prolonged fasting.

### A model of ANXA10 release dependent on sensing changes in gastric pH

The reproducible, secretion-like response of ANXA10 plasma levels during an OGTT was the most prominent formerly unreported finding of our study. Although identified in a glucose challenge, we believe that the robust response of ANXA10 arises from gastric processes rather than glucose metabolism per se. Convergent evidence from plasma proteomics, genetics, medication effects and tissue expression supports a model in which ANXA10 is released into the circulation from the stomach in response to increased gastric acid pH. During the OGTT, or after eating or drinking, the stomach pH increases due to dilution of the stomach acid [30]. Similarly, PPIs increase stomach pH by blocking acid secretion from parietal cells (Fig. 5a).

Sensing of stomach pH by the gastric epithelium is subject to a buffering effect of the mucous layer separating cells from the highly acidic stomach lumen [38]. Mucin 1 (MUC1), a key component of this mucous layer, forms a glycosylated extracellular domain with a tandem repeat region. We hypothesise that the genetically induced expansion of this region conferred by the *trans*-pQTL [39] results in a denser mucous layer [40, 41], with two relevant but independent consequences: providing better protection from *H. pylori* and gastric cancer, and altering epithelial pH sensing and thereby persistently increasing circulating ANXA10 levels (Fig. 5b). Such a change in pH sensing but not gastric acid pH per se is in line with a missing association between the gastric cancer risk variant (rs2990223, *MUC1*) and plasma secretin levels in UK Biobank (*p*=0.83), despite strong associations with other stomach-specific proteins (ESM Fig. 4d).

We note that our collective work on ANXA10 did not provide evidence for a role of ANXA10 in beta cell calcium handling and insulin secretion as proposed previously in mice [17, 42].

### Early responders to glucose ingestion

In addition to known early responders to glucose intake, such as GLP-1, we observed some less well understood candidates that may relate to glucose homeostasis and insulin secretion. Phogrin, for example, is highly expressed in pancreatic beta cells as part of insulin secretory granules, and has been suggested to regulate autocrine insulin signalling by binding to the activated insulin receptor to promote its dephosphorylation by protein tyrosine phosphatase 1B [43]. Further, while there is no clear mechanistic link to glucose homeostasis, higher plasma levels of SPOCK1 have been linked to slower type 2 diabetes progression [44].

### Effect of prolonged fasting on the OGTT response

Despite the major metabolic rearrangement resulting from prolonged fasting, key metabolic regulators, including GIP, GLP-1, fibroblast growth factor 21 (FGF21) [45] and ghrelin (Fig. 2b, ESM Fig. 2d), remained similarly responsive to glucose, findings that are in line with early work [46].

Only a few proteins showed altered responses, including some that are of potential relevance to insulin signalling and/or glucose homeostasis. Common genetic variation, probably acting via WARS, has been identified as contributing to fasting glucose levels [15, 36], and experimental work hypothesised a role for WARS in insulin resistance by tryptophanylation of the insulin receptor, although under conditions of excess tryptophan [47]. Our new observation linking the same genetic variation to the onset of coronary artery disease reiterates the need to elucidate the underlying mechanism. Further, the differential effect of motilin (Fig. 6a) provides new insights into hunger signalling following prolonged periods of fasting. Briefly, a decrease in plasma motilin levels during the OGTT is in line with the results of previous studies, and is specific to glucose ingestion (as opposed to mixed meals or lipid-rich meals) [35, 48]. Fluctuations of plasma motilin levels have further been shown to correlate with hunger scores that could be reproduced with erythromycin A, a motilin receptor agonist [49].

### Limitations and future directions

Although our study features a tightly controlled design, the small sample size did not permit us to test more moderate differences in plasma protein trajectories during the OGTT, including sex-differential trajectories, or associations between protein changes and the area under the curve for insulin and glucose. The repeated-measures design meant that our study was better powered to detect consistent rather than differential responses across both OGTTs. Both OGTTs were performed at the same time of the day, but we cannot fully exclude diurnal or other non-glucose effects on the proteins as we did not include participants who did not undergo an OGTT. All participants were healthy, and we were therefore unable to investigate the relevance of our findings among people with deteriorated metabolism, such as people with type 2 diabetes or gastric conditions. Further studies in vulnerable populations are needed to demonstrate the transferability of our findings. The unique ability of our study to profile the systemic response in humans complicated experimental follow-up studies, as it is not clear to what extent those mechanisms are preserved in model organisms. For example, loss of maternal *ANXA10* (or *Anxa10*) was associated with reduced litter size and embryonic mortality in mice and cattle [50] that otherwise appeared to be unimpaired, although gastric function and vulnerability to infections were not systematically tested for. Additionally, we inferred much regarding the tissue and cell type origins of ANXA10 that contribute to plasma levels from mRNA measurements, which do not always correlate with protein abundance, and further validation is needed at the protein level.

### Conclusion

Our study showed that the plasma proteome retains a dynamic response to nutrient intake even after 7 days of water-only fasting, largely originating from gastrointestinal tissues and possibly a result of evolutionary adaptations to prolonged periods without food.

## Supplementary Information

Below is the link to the electronic supplementary material.

ESM (PDF 5.22 MB)

ESM Tables (XLSX 4.18 MB)

## Data Availability

This work uses data provided by patients and collected by the NHS as part of their care and support. Individual-level data from UK Biobank are publicly available to bona fide researchers upon application (https://www.ukbiobank.ac.uk/).

## Abbreviations

ANXA10: Annexin A10
GIP: Gastric inhibitory polypeptide
GLP-1: Glucagon-like peptide-1
OGTT: Oral glucose tolerance test
pLOF: Predicted loss-of-function
PPI: Proton pump inhibitor
pQTL: Protein quantitative trait loci
WARS: Tryptophanyl-tRNA synthetase 1

## Code availability

The code used to generate the results and figures in this study is available at https://github.com/comp-med/olink-ogtt.
