# Supplementary material for: Tissue origins of the plasma proteomic response to glucose ingestion in humans
Source: Diabetologia. 2026 Jul 17;69(10):2970–82. doi: 10.1007/s00125-026-06800-8 (PMC13541793; doi:10.1007/s00125-026-06800-8)
Supplement: Supplementary file 1 [file 125_2026_6800_MOESM1_ESM.pdf]

ESM Methods

Study design

We performed OGTTs (75 g glucose dissolved in 300 ml water) on 11 apparently healthy, normoglycaemic, non-smoking, self-reported European participants at two timepoints: after an overnight fast, and after a 7 day diet allowing only ad libitum water intake (Fig. 1a). We refer to these subsequently as 'OGTT1' and 'OGTT2', respectively. The exclusion criteria were any known disease and percentage body fat below 15% for female participants and 12% for male participants. To examine potential sex-differential effects we included five women and six men for whom self-report agreed with sex-specific proteomic profiles. Participant characteristics are shown in ESM Table 1. We have already demonstrated good adherence of participants to the fasting scheme in the absence of adverse events [1], but note that one female participant did not complete OGTT2. During both OGTTs, we sampled blood from each participant at the start as well as after 15, 30, 60 and 120 min following glucose ingestion for time-resolved proteomic profiling. Plasma insulin and glucose were measured as described elsewhere [2].

All participants gave their written informed consent, and the study was approved by the local ethics committee (Norwegian School of Sport Sciences, no. #15-220817).

Proteomics measurements

Using the Olink 3072 Explore platform we measured the plasma levels of 2923 unique protein targets across 2941 assays in 105 samples. Olink technology[3, 4] and quality control details[1] have been described previously. During QC, we removed data from one panel (Cardiometabolic; OGTT2: minute 60) for one sample, and from three panels (Neurology, Neurology_II, Oncology; OGTT1; minute 0) for another sample due to outlier protein levels, following Olink's guidance on handling such cases[5]. One female participant did not have OGTT2 data. Figures and text that refer to GLP-1 are based on the Olink assay "GCG", which likely does not distinguish glucagon and GLP-1, and the trajectory during the OGTT suggests the signal is GLP-1.

Statistical analysis of glucose, insulin, and protein trajectories

We tested for time-varying effects during each OGTT, differential effects across OGTTs, and sex-differential effects on insulin, glucose, and protein levels before and after fasting using linear mixed models. We used z-score standardized NPX values for each protein using the mean and standard deviation at minute 0 of OGTT1, such that values represent SD units relative to OGTT1 minute 0.

We implemented linear mixed models using the R package *lmerTest* (v3.1.3)[6], with the timepoint as fixed effect and participant as random effect. To test for potential sex- or fasting status-differential effects, we additionally included a 'time x sex' or 'time x OGTT session' interaction term in the model. We considered only sex differential effects that showed evidence across both OGTTs (i.e. passing multiple testing correction in one and at least nominal significance in the other OGTT). To test whether proteomic changes associated with those of plasma glucose and insulin levels on each OGTT, we included glucose or insulin as the fixed effect and participant and timepoint as random effects.

For time varying effects during each OGTT, we calculated a global p-value including all timepoints using an analysis of variance (ANOVA) implemented by aov() function. We used the Benjamini-Hochberg procedure to control the false discovery rate (q-value) at 5% across all proteins, except for the interaction analysis, where we used a 20% threshold due to lower power than main effects.

We added median NPX per sample and panel as a fixed effect when analysing protein levels because we observed varying median NPX values across timepoints especially in OGTT2. Sensitivity analysis with and without including this covariate showed similar results, with attenuated p-values upon inclusion in the model.

We performed several sensitivity checks (including or excluding median sample NPX as a covariate, and normalizing all NPX values to day 0 minute 0 or to minute 0 on each day), and report only those proteins that passed each of these checks as "robust" signals for a differential effect.

Insulin indices

We calculated surrogate measures of insulin sensitivity using fasting glucose and insulin concentrations across the days of the fast. We used HOMA-IR (Homeostatic Model Assessment for Insulin Resistance), HOMA-B (Homeostasis Model Assessment of Beta-cell function)[7], and QUICKI (quantitative insulin sensitivity check index)[8].

We further tested indices based on previously established models to estimate insulin sensitivity and beta cell function from glucose and insulin concentrations during an OGTT. We included Stumvoll MCR (metabolic clearance rate), Stumvoll ISI (insulin sensitivity index), Stumvoll first- and second-phase insulin release[9], insulin fold change, HIRI (hepatic insulin resistance index)[10, 11], (incremental) area under the glucose curve[12], Matsuda[13], and insulinogenic index. Stumvoll indices have alternative formulas based on availability of measurements on different time points, and where applicable we tested multiple ones.

Calculations we used are listed below. Units are in parentheses, and subscripts show timepoint of the OGTT.

HOMA-IR = fasting glucose (mmol/l) * fasting insulin (mU/l) / 22.5
HOMA-B = [20 * fasting insulin (mU/l)] / [fasting glucose (mmol/l) - 3.5]
QUICKI = 1 / (log10(fasting insulin mU/ml) + log10(fasting glucose mg/dl))
(The Stumvoll formulas use insulin in pmol/l, glucose in mmol/l)
Stumvoll ISI $_{0 \text{ and } 120}$ = 0.156 - 0.0000459 * insulin_{120} - 0.000321 * insulin_{0} - 0.00541 * glucose_{120}
Stumvoll ISI $_{0 \text{ and } 60}$ = 0.149 - 0.000467 * insulin_{0} - 0.00466 * glucose_{60}
Stumvoll MCR $_{0 \text{ and } 120}$ = 13.273 - 0.00384 * insulin_{120} - 0.0232* insulin_{0} - 0.463 * glucose_{120}
Stumvoll MCR $_{0 \text{ and } 60}$ = 12.464 - 0.0357* insulin_{0} - 0.376 * glucose_{60}
Stumvoll first phase $_{0 \text{ and } 30}$ = 1283 + 1.829 * insulin_{30} - 138.7 * glucose_{30} + 3.772 * insulin_{0}
Stumvoll first phase $_{0 \text{ and } 60}$ = 1194 + 4.724* insulin_{0} - 117 * glucose_{60} + 1.414 * insulin_{60}
Stumvoll second phase $_{0 \text{ and } 30}$ = 286 + 0.416 * insulin_{30} - 25.94 * glucose_{30} + 0.926 * insulin_{0}
Stumvoll second phase $_{0 \text{ and } 60}$ = 295 + 0.349 * insulin_{60} - 25.72 * glucose_{60} + 1.107 * insulin_{0}
Insulin fold change = ln(insulin_{120} / insulin_{0})
Glucose $\text{AUC}_{0\text{-}120}$ was estimated using the trapezoidal rule. Incremental glucose AUC was estimated until the first glucose nadir (if reached before minute 120), by subtracting the basal glucose rectangle from the AUC until the nadir timepoint.
HIRI = Glucose $\text{AUC}_{0\text{-}30}$ (mg/min^{-1}/dl) * Insulin $\text{AUC}_{0\text{-}30}$ (μU/min^{-1}/ml)

$Matsuda = 10,000 / \sqrt{[glucose_0 (mg/dl) * insulin_0 (mU/L) * mean\ glucose * mean\ insulin]}$

$Insulinogenic\ index = [insulin_{30} - insulin_0 (pmol/L)] / [glucose_{30} - glucose_0 (mmol/L)]$

Tissue enrichment analysis

We programmatically retrieved tissue specificity data for the Olink proteins in JSON format from the Human Protein Atlas (HPA, https://www.proteinatlas.org/) on 24.07.2023, which was available for all except 5 (ANP32C, BTNL10, KIR2DL2, LILRA3, PALM2AKAP2). For proteins that were classified as "Tissue enriched" or "Tissue enhanced" by HPA, we assigned them to the tissue with the maximum nTPM value to define tissue specificity. We then performed a one-sided Fisher's exact test to determine whether proteins specifically expressed in a tissue were enriched among those that responded in either OGTT.

Participant characteristics and trans-pQTL enrichment analyses

We used the enrichment_test_across_groups() function from the prodente v0.2.0 package to evaluate enrichment of protein signatures associated with various aspects of human health and disease[14]. To explore regulatory origins, OGTT signatures were tested for overrepresentation of proteins linked to one or more of 652 pleiotropic protein quantitative trait loci[15] (pQTL; associated with ≥5 proteins per locus), grouping pQTLs in high LD (r^2>0.7) and assigning candidate effector genes according to Sun et al [15].

Genetic and transcriptomic analyses

Genome-wide association study

We conducted a genome-wide association study (GWAS) of ANXA10 plasma levels in 46,929 European ancestry participants in UK Biobank. We defined genetic ancestry based on the Pan-UKB definition[16], and further assigned European participants not classified by the Pan-UKB team based on a k-means neighbour approach using the first four genetic principal components to minimize data loss. We used inverse rank normalized Olink protein abundances as the outcome. Age, sex, centre, batch, first 10 principal components, and technical covariates (sample age, month and time of blood draw, hours of fasting) were included as covariates. GWAS was performed using REGENIE v3.2.5[17] in a two-step approach as implemented by

the software. In the first step, we used genotyped single nucleotide polymorphisms (SNPs) meeting quality control (QC) criteria of minor allele frequency (MAF) >1%, minor allele count (MAC) >100, Hardy-Weinberg equilibrium (HWE) p-value $>1\times10^{-15}$, and genotype missingness rate <1%, excluding individuals with more than 10% missing genotype data. We pruned these variants for linkage disequilibrium (LD) with a window of 1,000 variants, a sliding window of 100, and an r^2 threshold of <0.8, leaving 481,526 variants. We tested ~15 million imputed variants that passed filters of MAF>0.001 and HWE p-value$>1\times10^{-15}$ in the second step. The loci were considered genome-wide significant if 2 or more variants passed the genome-wide significance threshold ($5x10^{-8}$), to avoid spurious associations.

Genetic colocalization analyses

We assessed whether ANXA10 and WARS pQTLs (rs4600882, rs2990223, rs2273804) influenced gene expression across 49 GTEx v8[18] tissues through colocalization using the coloc R package[19] (v5.2.3). At the ANXA10 pQTL in the *MUC1* locus, only a splicing QTL was detected for *MUC1* in GTEx, and we therefore used a stomach dataset to further explore stomach-specific eQTLs for *ANXA10* and *MUC1*. The dataset from Hess et al[20] complements GTEx by offering eQTL data from living donors, and coverage of both corpus and antrum regions. We tested ANXA10 pQTLs for colocalization with risk variants shown for gastric cancer (Hess et al.[20]) and gastric polyp (Sakaue et al.[21]) in the same region. We further tested *cis*-protein colocalization (gene body ± 500kb) of pQTLs (from Sun et al.[15]) for any OGTT-responsive proteins with 30 endpoints including 26 cardiovascular outcomes as described previously[22] and four glycemic traits[23]. Summary statistics were preprocessed using MungeSumStats[24]. We formally tested for colocalisation in 500kb regions around sentinel pQTLs when the endpoint reached suggestive statistical evidence in that same region (p-value $< 5 \times 10^{-6}$). For colocalization analyses, we set $p_{12}=5x10^{-6}$, and a posterior probability of ≥ 75% was considered evidence for a shared genetic signal.

Single-cell expression profiling

Single-cell RNA-sequencing data from gastrointestinal[25] and pancreatic[26] tissues were analysed with default Seurat[27] (v5.0.3) functions to visualize ANXA10 expression. We calculated the Spearman correlation of each gene with *ANXA10* in all healthy gastrointestinal or pancreatic cells and defined co-expression as the top 5th percentile of all correlations. For the pancreas feature plot, we rearranged the cells to place the ones with higher ANXA10 expression on top due to the low number of cells with expression.

To compare putative protein interaction partners of *ANXA10* against the coexpression pattern, we queried ANXA10 in STRING[28] v12.0 (default settings plus up to five interactors in the second shell).

Phenotypic associations with plasma protein levels in UK Biobank

In 52,140 UK Biobank participants with Olink data, we evaluated the influence of prevalent gastric diseases and intake of acid-suppressing drugs on protein levels using linear models adjusted for age and sex.

Gastric disease ascertainment

To identify UK Biobank participants with different existing gastric diseases at baseline, we used phecodes that we compiled from ICD-coded self-reports, hospital episode statistics, cancer registry, and primary care records (available for 45% of the cohort). Records with diagnosis dates before or within the participant's birth year were excluded to minimize potential coding errors in electronic health records. Diagnoses were then mapped to 1,106 distinct 'phecodes' that represent medical ontology terms to reduce redundancy across ICD-10 codes[29]. For participants with phecodes for multiple gastric diseases, we considered the most recent occurring diagnosis before the baseline visit. We excluded participants that had gastrectomy before baseline, since this would impact plasma levels of proteins with stomach origin. We also excluded participants with first incident gastric disease after baseline, due to uncertainty whether disease occurred but was not diagnosed before baseline. We then grouped phecodes into categories of similar disease, identifying 3731 participants with gastroesophageal reflux disease (GERD), reflux, or esophagitis; 1165 with diaphragmatic hernia; 884 with peptic ulcer; 1398 with gastritis; 235 with Barrett's oesophagus or ulcer of oesophagus; 30 with stomach or oesophageal cancer; 14 with atrophic gastritis before the baseline visit who had Olink data (out of 52,140 in total).

To identify participants seropositive for *H. pylori*, we used the "H. pylori Definition II" field (field id 23074), identifying 317 participants who were seropositive and had Olink data, out of 985 in total.

Acid lowering medication ascertainment

To identify participants taking acid lowering medication at the baseline visit, we obtained mappings of self-reported brand names to ATC codes reported previously[30]. We used the ATC codes starting with A02BC for PPIs and A02BA for H_2-receptor antagonists (H2RA). We identified 6462 participants taking acid lowering medication (5655 for PPI, 985 for H2RA) that had Olink data in UK Biobank.

For a subset of our UKB study population (n=230,041) linked electronic health records to general practitioner (GP) visits and associated drug prescriptions were available. We collated information on PPI prescription by grepping all listed drug names containing 'omeprazole', 'esomeprazole', 'lansoprazole', 'pantoprazole', or 'rabeprazole', with available dose information resulting in a total of 2,825,996 prescriptions issued between 1962 and 2017 for a total of 107,412 participants. To demonstrate dosage-dependent effects, we defined PPI usage based on the prescription most recently issued but not longer than 3 months apart from the baseline examination. We identified participants with ≥2 prescriptions both before and after their baseline visit, with the most recent prescription less than 3 months before baseline as chronic PPI users (n=1915).

Gastrectomy ascertainment

We identified participants who underwent gastrectomy before the baseline visit using OPCS4 codes starting with G27 (total gastrectomy) or G28 (partial gastrectomy) from Health Episode Statistics records (hesin_oper table) and identified 14 such participants with Olink data. We did not perform linear regression here due to the low number of participants.

All the research in UK Biobank was conducted under Application Number 44448.

Protein association profile of gastrointestinal health SNPs

To systematically profile the proteomic impact of SNPs linked to gastrointestinal health, including the ANXA10 trans-pQTL (rs2990223), we generated a list of independent SNPs associated with gastric phenotypes and were cis- or trans-pQTLs for at least one protein, based on our previous work[14]. We excluded proteins that had only a trans-pQTL in the *ABO* region, leaving 7 loci. We then extracted summary statistics for these SNPs in the UKB-PPP summary statistics from Sun et al, across 42 proteins[15].

ANXA10 pLOF carrier in Genes & Health cohort

Genes & Health is a community based study of British Pakistani and British Bangladeshi people aged 16 or older living in the UK[31]. Approximately 44,028 individuals have whole exome sequencing and National NHS and primary and secondary care electronic health record linkage to date. We conducted this research under an approved application to the Genes & Health Executive. The Genes & Health study is approved by the London South East NRES Committee of the Health Research Authority (14/LO/1240). Whole exome sequencing data was generated using Twist Clinical Research Alliance reagent and 150bp paired end high depth sequencing. The generation and quality control of exome sequencing data, statistical phasing, and the extraction and preparation of phenotypes has been previously described[32]. Variants were annotated using the Ensembl Variant Effect Predictor (VEP v105) with LOFTEE plugin (v1.04_GRCh38), as previously described[32].

We assessed the presence of individuals homozygous for predicted loss of function (pLOF; premature stop, indels, splicing) variants within ANXA10. We identified a single carrier of a homozygous pLOF variant within ANXA10: chr4:168164289:G>A (rs752829627). Disease diagnoses and the date of diagnosis were extracted from linked EHR records coded as ICD10 codes. All diagnostic codes were assessed to ensure that no codes had unrealistic timestamps (e.g. before or on date of birth or after the date of last data extraction). For this individual all present diagnostic codes passed these QC criteria. To protect the identity of the individual no specific dates or age of diagnosis for reported conditions, or the location recruitment site are reported within this publication.

REFERENCES

1. Pietzner M, Uluvar B, Kolnes KJ, et al (2024) Systemic proteome adaptions to 7-day complete caloric restriction in humans. Nat Metab 6(4):764–777. https://doi.org/10.1038/s42255-024-01008-9

2. Rustad PI, Sailer M, Cumming KT, et al (2016) Intake of Protein Plus Carbohydrate during the First Two Hours after Exhaustive Cycling Improves Performance the following Day. PloS One 11(4):e0153229. https://doi.org/10.1371/journal.pone.0153229

3. Assarsson E, Lundberg M, Holmquist G, et al (2014) Homogenous 96-plex PEA immunoassay exhibiting high sensitivity, specificity, and excellent scalability. PloS One 9(4):e95192. https://doi.org/10.1371/journal.pone.0095192

4. Zhong W, Edfors F, Gummesson A, Bergström G, Fagerberg L, Uhlén M (2021) Next generation plasma proteome profiling to monitor health and disease. Nat Commun 12(1):2493. https://doi.org/10.1038/s41467-021-22767-z

5. Nevola K, Sandin M, Guess J, et al (2022) OlinkAnalyze: Facilitate Analysis of Proteomic Data from Olink. 4.2.0

6. Kuznetsova A, Brockhoff PB, Christensen RHB (2017) **lmerTest** Package: Tests in Linear Mixed Effects Models. J Stat Softw 82(13). https://doi.org/10.18637/jss.v082.i13

7. Matthews DR, Hosker JP, Rudenski AS, Naylor BA, Treacher DF, Turner RC (1985) Homeostasis model assessment: insulin resistance and ?-cell function from fasting plasma glucose and insulin concentrations in man. Diabetologia 28(7):412–419. https://doi.org/10.1007/BF00280883

8. Katz A, Nambi SS, Mather K, et al (2000) Quantitative insulin sensitivity check index: a simple, accurate method for assessing insulin sensitivity in humans. J Clin Endocrinol Metab 85(7):2402–2410. https://doi.org/10.1210/jcem.85.7.6661

9. Stumvoll M, Van Haeften T, Fritsche A, Gerich J (2001) Oral Glucose Tolerance Test Indexes for Insulin Sensitivity and Secretion Based on Various Availabilities of Sampling Times. Diabetes Care 24(4):796–797. https://doi.org/https://doi.org/10.2337/diacare.24.4.796

10. Muniyappa R, Tella SH, Sortur S, et al (2019) Predictive Accuracy of Surrogate Indices for Hepatic and Skeletal Muscle Insulin Sensitivity. J Endocr Soc 3(1):108–118. https://doi.org/10.1210/js.2018-00206

11. Williamson A, Norris DM, Yin X, et al (2023) Genome-wide association study and functional characterization identifies candidate genes for insulin-stimulated glucose uptake. Nat Genet 55(6):973–983. https://doi.org/10.1038/s41588-023-01408-9

12. Floch J-PL, Escuyer P, Baudin E, Baudon D, Perlemuter L (1990) Blood Glucose Area Under the Curve: Methodological Aspects. Diabetes Care 13(2):172–175. https://doi.org/10.2337/diacare.13.2.172

13. Matsuda M, DeFronzo RA (1999) Insulin sensitivity indices obtained from oral glucose tolerance testing: comparison with the euglycemic insulin clamp. Diabetes Care 22(9):1462–1470. https://doi.org/10.2337/diacare.22.9.1462

14. Pietzner M, Beuchel C, Demircan K, et al (2025) Machine learning-guided deconvolution of plasma protein levels. Mol Syst Biol 21(12):1822–1844. https://doi.org/10.1038/s44320-025-00158-6

15. Sun BB, Chiou J, Traylor M, et al (2023) Plasma proteomic associations with genetics and health in the UK Biobank. Nature 622(7982):329–338. https://doi.org/10.1038/s41586-023-06592-6

16. Karczewski KJ, Gupta R, Kanai M, et al (2025) Pan-UK Biobank genome-wide association analyses enhance discovery and resolution of ancestry-enriched effects. Nat Genet 57(10):2408–2417. https://doi.org/10.1038/s41588-025-02335-7

17. Mbatchou J, Barnard L, Backman J, et al (2021) Computationally efficient whole-genome regression for quantitative and binary traits. Nat Genet 53(7):1097–1103. https://doi.org/10.1038/s41588-021-00870-7

18. The GTEx Consortium, Aguet F, Anand S, et al (2020) The GTEx Consortium atlas of genetic regulatory effects across human tissues. Science 369(6509):1318–1330. https://doi.org/10.1126/science.aaz1776

19. Giambartolomei C, Vukcevic D, Schadt EE, et al (2014) Bayesian test for colocalisation between pairs of genetic association studies using summary statistics. PLoS Genet 10(5):e1004383. https://doi.org/10.1371/journal.pgen.1004383

20. Hess T, Maj C, Gehlen J, et al (2023) Dissecting the genetic heterogeneity of gastric cancer. eBioMedicine 92:104616. https://doi.org/10.1016/j.ebiom.2023.104616

21. Sakaue S, Kanai M, Tanigawa Y, et al (2021) A cross-population atlas of genetic associations for 220 human phenotypes. Nat Genet 53(10):1415–1424. https://doi.org/10.1038/s41588-021-00931-x

22. Zoodsma M, Beuchel C, Yasmeen S, et al (2025) A genetic map of human metabolism across the allele frequency spectrum. Nat Genet 57(10):2445–2455. https://doi.org/10.1038/s41588-025-02355-3

23. Chen J, Spracklen CN, Marenne G, et al (2021) The trans-ancestral genomic architecture of glycemic traits. Nat Genet 53(6):840–860. https://doi.org/10.1038/s41588-021-00852-9

24. Murphy AE, Schilder BM, Skene NG (2021) MungeSumstats: a Bioconductor package for the standardization and quality control of many GWAS summary statistics. Bioinforma Oxf Engl 37(23):4593–4596. https://doi.org/10.1093/bioinformatics/btab665

25. Nowicki-Osuch K, Zhuang L, Cheung TS, et al (2023) Single-Cell RNA Sequencing Unifies Developmental Programs of Esophageal and Gastric Intestinal Metaplasia. Cancer Discov 13(6):1346–1363. https://doi.org/10.1158/2159-8290.CD-22-0824

26. Elgamal RM, Kudtarkar P, Melton RL, et al (2023) An Integrated Map of Cell Type–Specific Gene Expression in Pancreatic Islets. Diabetes 72(11):1719–1728. https://doi.org/10.2337/db23-0130

27. Hao Y, Hao S, Andersen-Nissen E, et al (2021) Integrated analysis of multimodal single-cell data. Cell 184(13):3573-3587.e29. https://doi.org/10.1016/j.cell.2021.04.048

28. Szklarczyk D, Kirsch R, Koutrouli M, et al (2023) The STRING database in 2023: protein-protein association networks and functional enrichment analyses for any sequenced genome of interest. Nucleic Acids Res 51(D1):D638–D646. https://doi.org/10.1093/nar/gkac1000

29. Bastarache L (2021) Using Phecodes for Research with the Electronic Health Record: From PheWAS to PheRS. Annu Rev Biomed Data Sci 4:1–19. https://doi.org/10.1146/annurev-biodatasci-122320-112352

30. Wu Y, Byrne EM, Zheng Z, et al (2019) Genome-wide association study of medication-use and associated disease in the UK Biobank. Nat Commun 10(1):1891. https://doi.org/10.1038/s41467-019-09572-5

31. Finer S, Martin HC, Khan A, et al (2020) Cohort Profile: East London Genes & Health (ELGH), a community-based population genomics and health study in British Bangladeshi and British Pakistani people. Int J Epidemiol 49(1):20–21i. https://doi.org/10.1093/ije/dyz174

32. Kim HI, DeBoever C, Walter K, et al (2025) Exome sequencing and analysis of 44,028 British South Asians enriched for high autozygosity

ESM Figure 1: Insulin indices tested that were altered by fasting

a

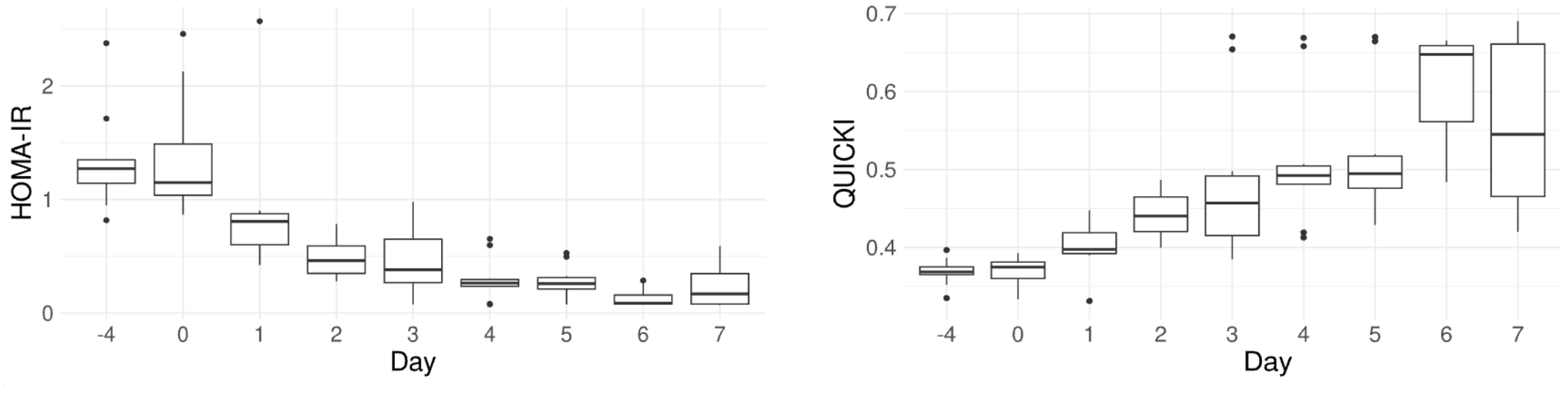

b

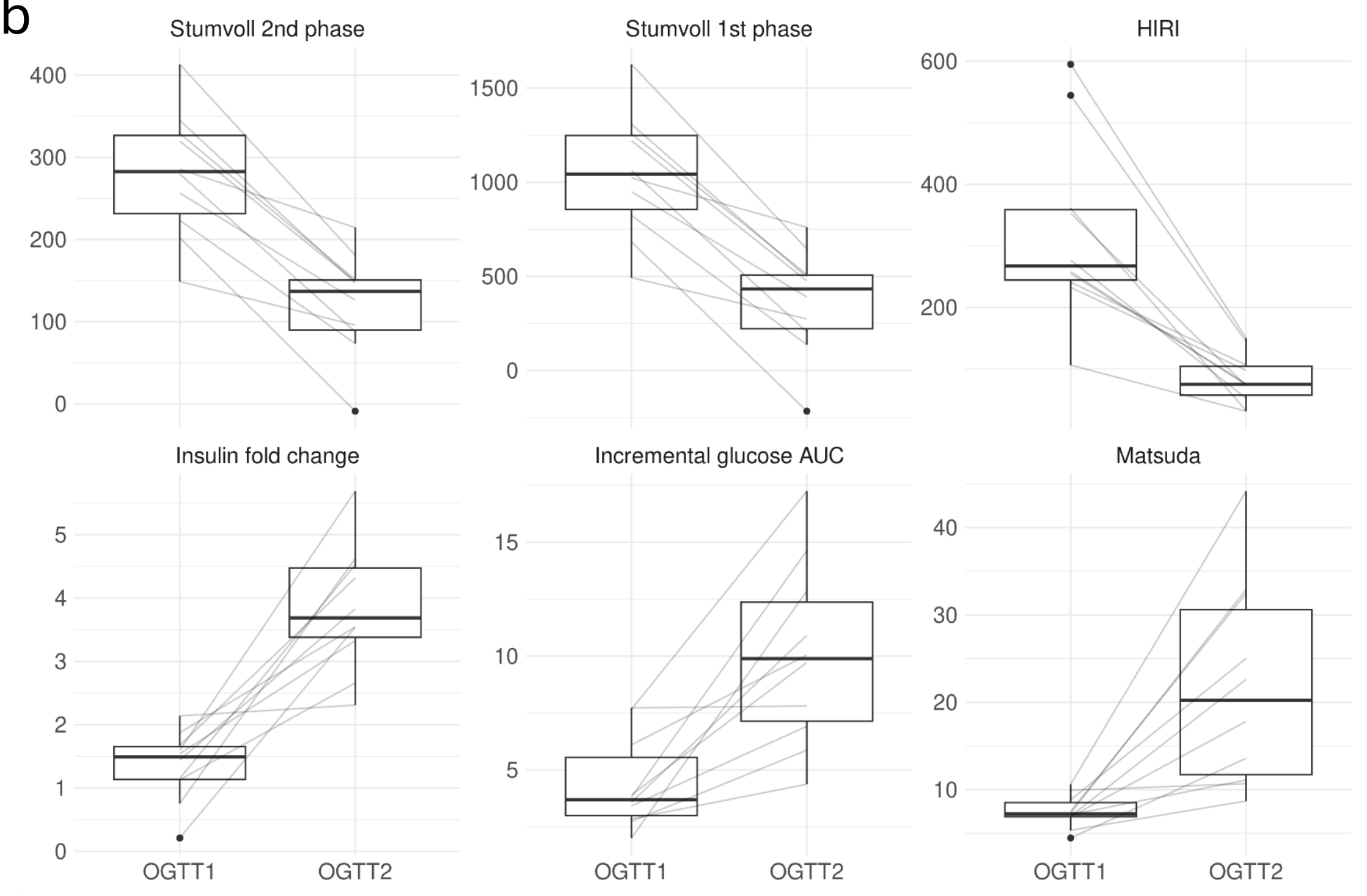

(a) Boxplots showing the HOMA-IR and QUICKI indices for insulin resistance and sensitivity, respectively, across the days of the experiment.

(b) Boxplots comparing OGTT-derived indices on OGTT 1 and 2: Stumvoll 2nd and 1st phase insulin release, Hepatic insulin resistance index (HIRI), insulin fold change from 0 to 120 minutes, incremental area under the glucose curve, and Matsuda insulin sensitivity index. Grey lines connect individual values. Boxes show the median and IQR, and the whiskers extend to the furthest point within 1.5×IQR.

ESM Figure 2: Plasma proteome response

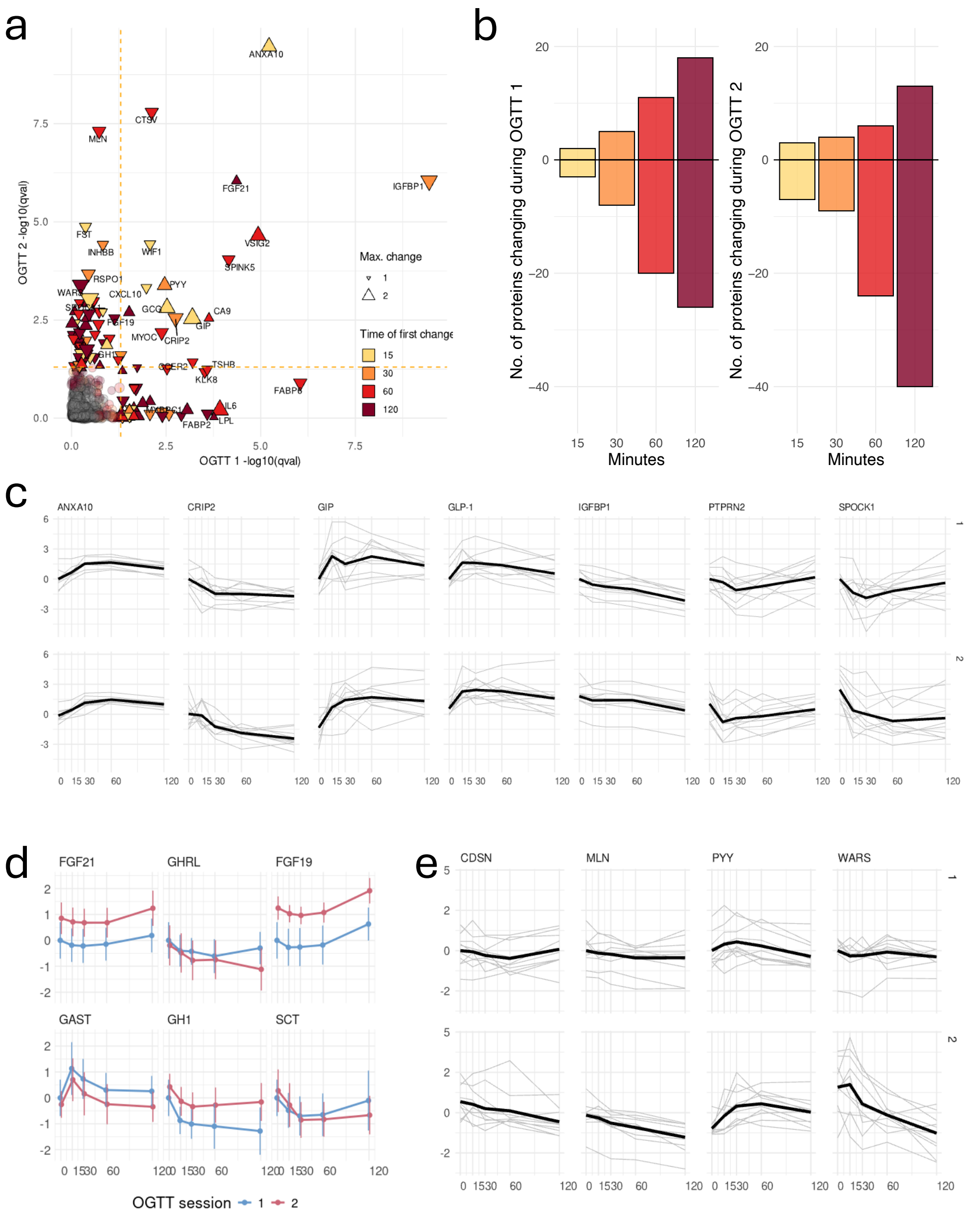

(a) Scatterplot of the significance level of each protein response in OGTT 1 vs OGTT 2. Colours indicate the first time point at which the protein was significantly different from minute 0. Direction of the triangles indicate whether the maximum change is positive or negative. Size of the triangles indicate the magnitude of the maximum change.

(b) Number of proteins that changed during the first (left) and the second (right) OGTT. Positive bars show the number of proteins that increase, and negative values show proteins that decrease, compared to minute 0 on each time point.

(c) Individual trajectories of the proteins in Figure 2b. Grey lines show individual participants and black line shows the mean. The top row is OGTT 1 and the bottom row is OGTT 2.

(d) Trajectories of additional proteins mentioned in the text. Error bars indicate 95% CIs.

(e) Individual trajectories of the proteins in Figure 6a.

ESM Figure 3: Tissue enrichment of OGTT-responsive proteins

a

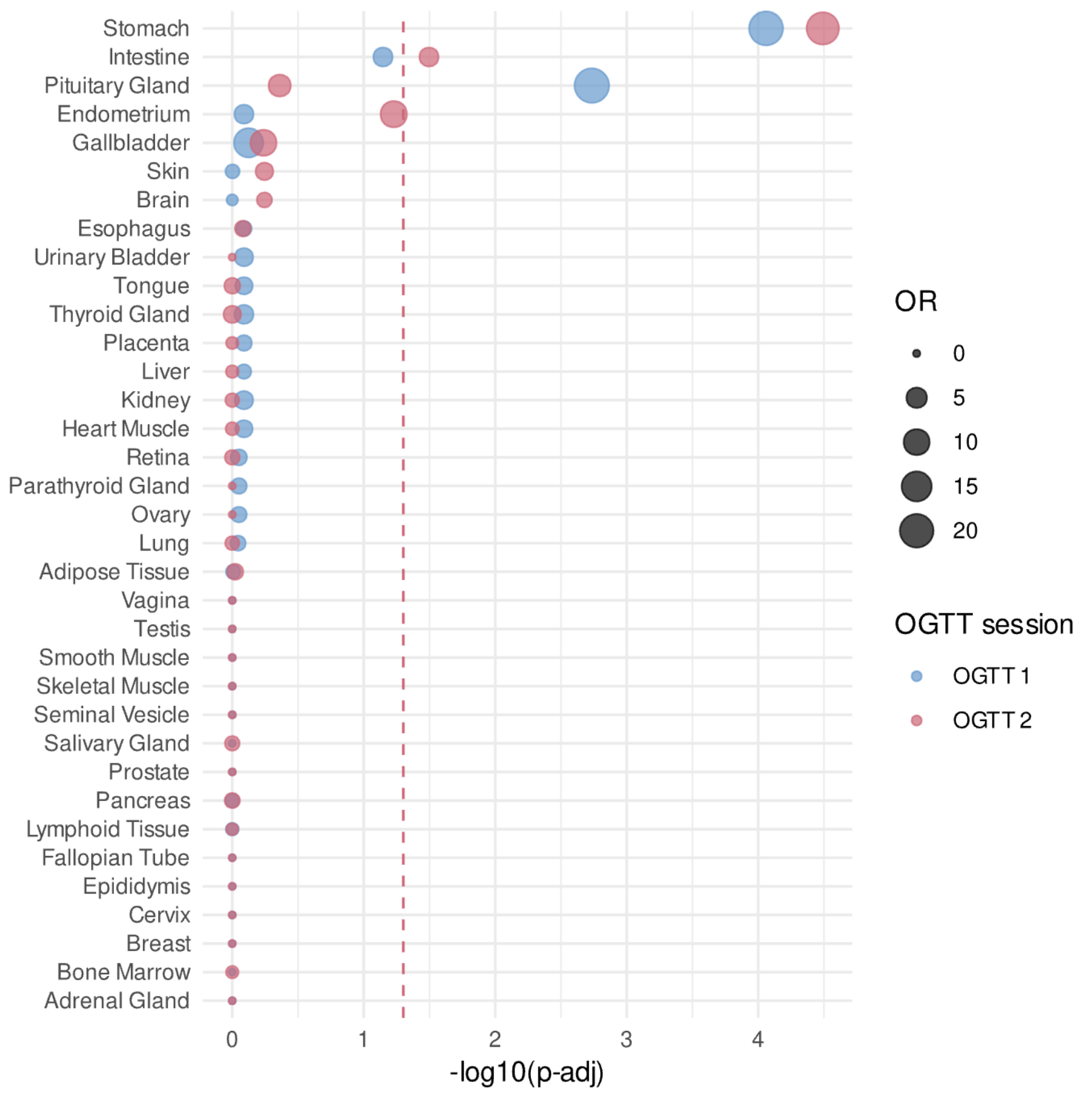

b

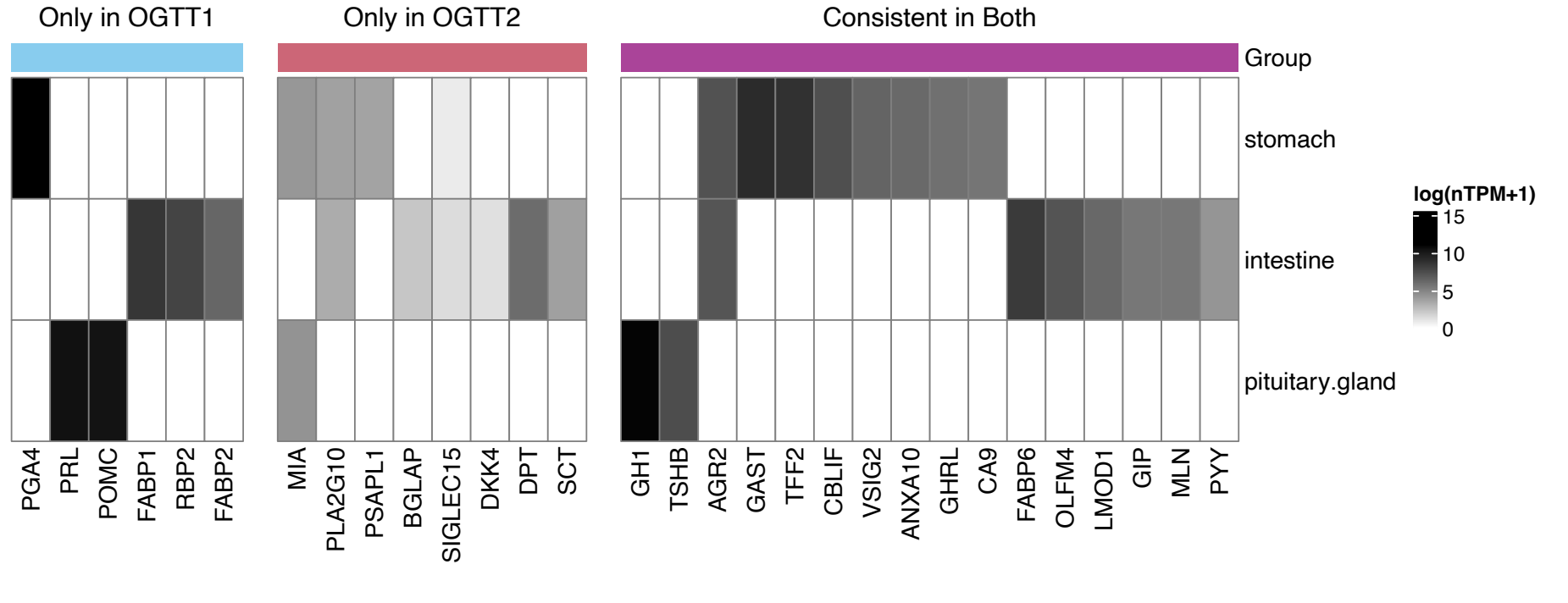

(a) Enriched tissues with specific expression of genes whose protein products change during the first or second OGTT, according to Human Protein Atlas (HPA).

(b) Heatmap of normalized mRNA expression values (nTPM) from HPA per gene per tissue, restricted to tissues and genes identified above and grouped by consistency of the OGTT response.

ESM Figure 4: Impact of proton pump inhibitors and gastric disease on protein levels in UK Biobank

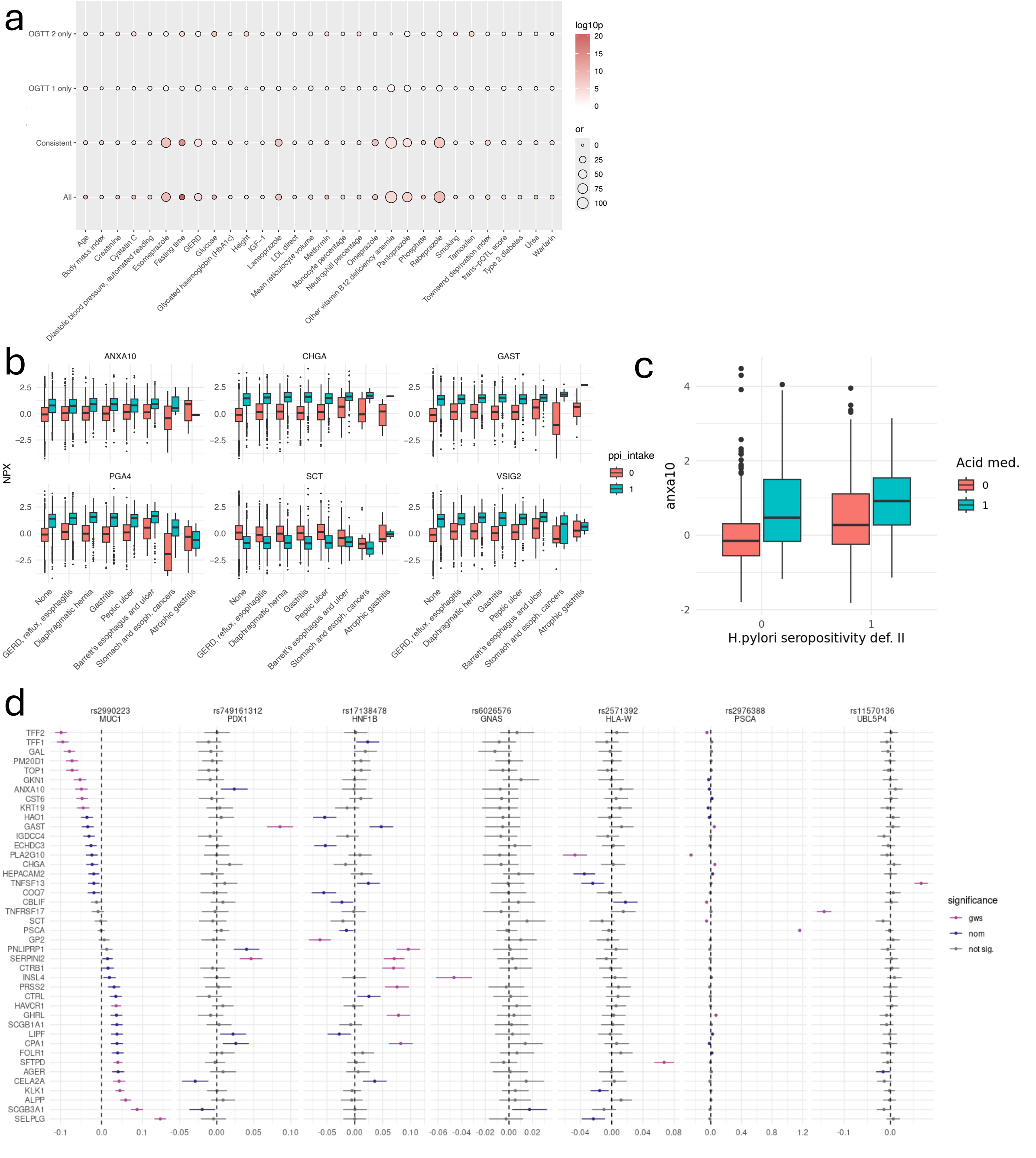

(a) Enrichment of protein signatures associated with participant characteristics among groups of OGTT-responsive proteins. The R package 'prodente' (https://github.com/comp-med/r-prodente) was used to perform the enrichment test and plot the results.

(b) INT-transformed NPX of selected proteins impacted by PPI intake, stratified by gastric disease type.

(c) INT-transformed NPX of ANXA10 levels in participants by *H. pylori* seropositivity (definition II) and PPI intake, from UKB infectious agent pilot study. Boxes show the median and IQR, and the whiskers extend to the furthest point within 1.5×IQR.

(d) Forest plot of the effects of seven gastric disease-associated SNPs across 42 proteins significantly associated with at least one. The bioinformatically annotated gene is shown under each SNP. gws=genome-wide significant, nom=nominally significant, not sig=not significant. Error bars indicate 95% CIs.

ESM Figure 5: Genetic associations with plasma ANXA10

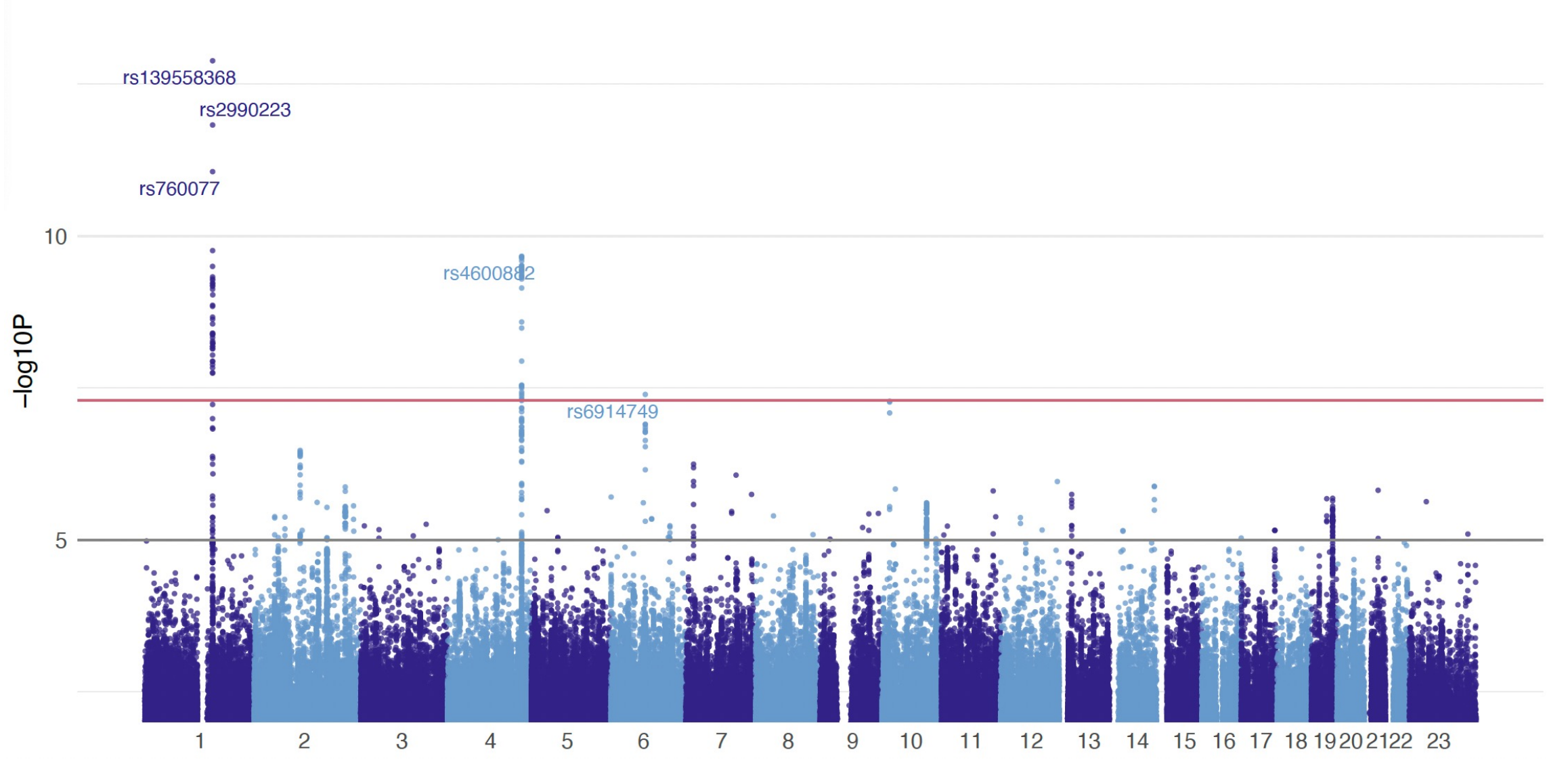

Manhattan plot of GWAS for plasma ANXA10 levels.

ESM Figure 6: Tissue origins of plasma ANXA10

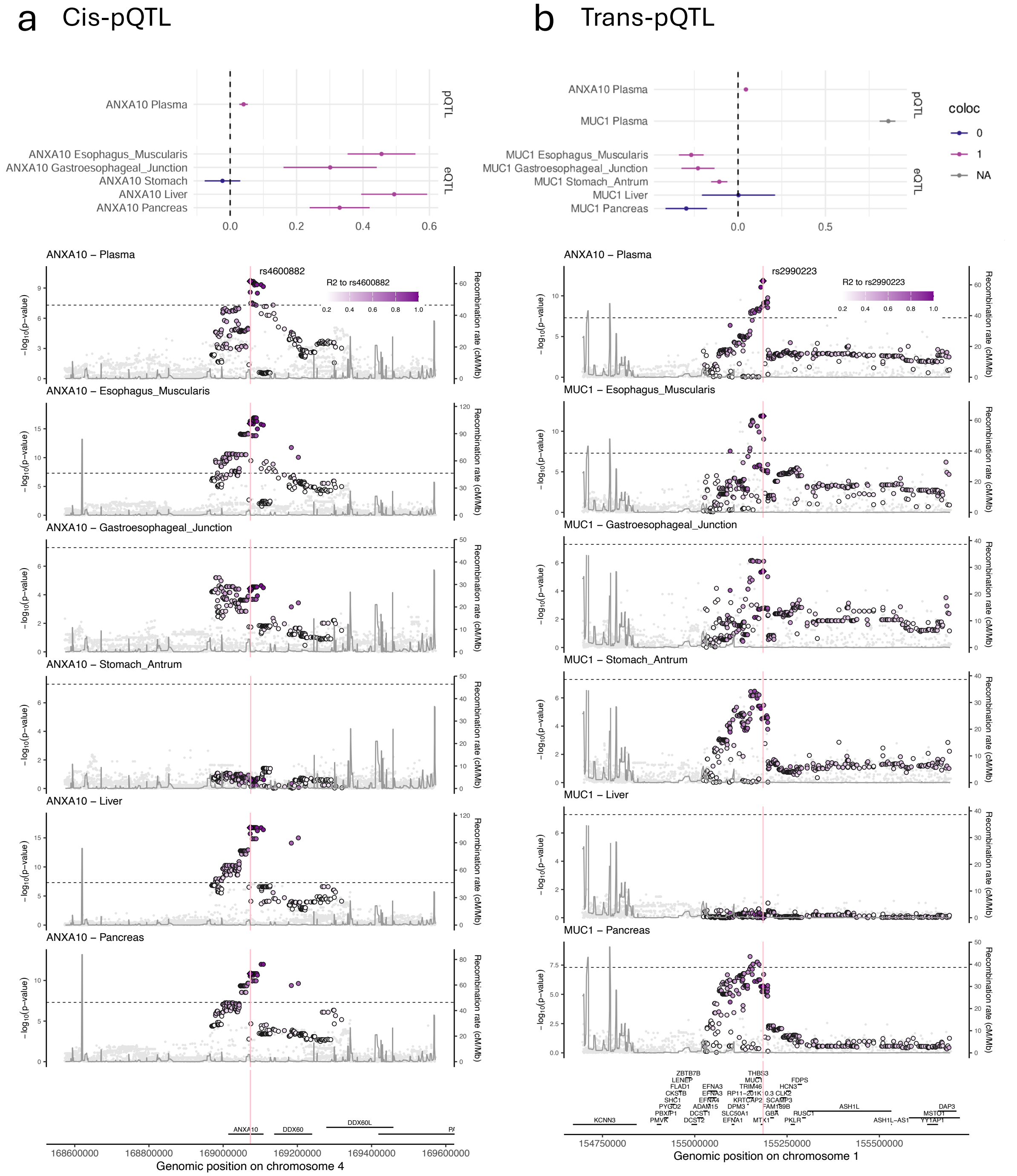

(top) Forest plots of the effect sizes (in s.d. units) of rs4600882 (left) and rs2990223 (right) on plasma protein and tissue mRNA levels. The color of the lines indicate presence or absence of colocalization (PP.H4>0.75). Error bars indicate 95% CIs.
(bottom) Regional association plots of 1 Mb region around the cis- (rs4600882, left) and trans-pQTL (rs2990223, right). Different plots show GWAS results for plasma ANXA10, tissue-level eQTLs for *ANXA10* and *MUC1* from a stomach-specific dataset (Hess et al, 2023), and GTEx (rest of the tissues shown). Only those tissues that colocalize with either the cis or trans signal are shown.

ESM Figure 7: Support for non-stomach tissue origins of plasma ANXA10

a

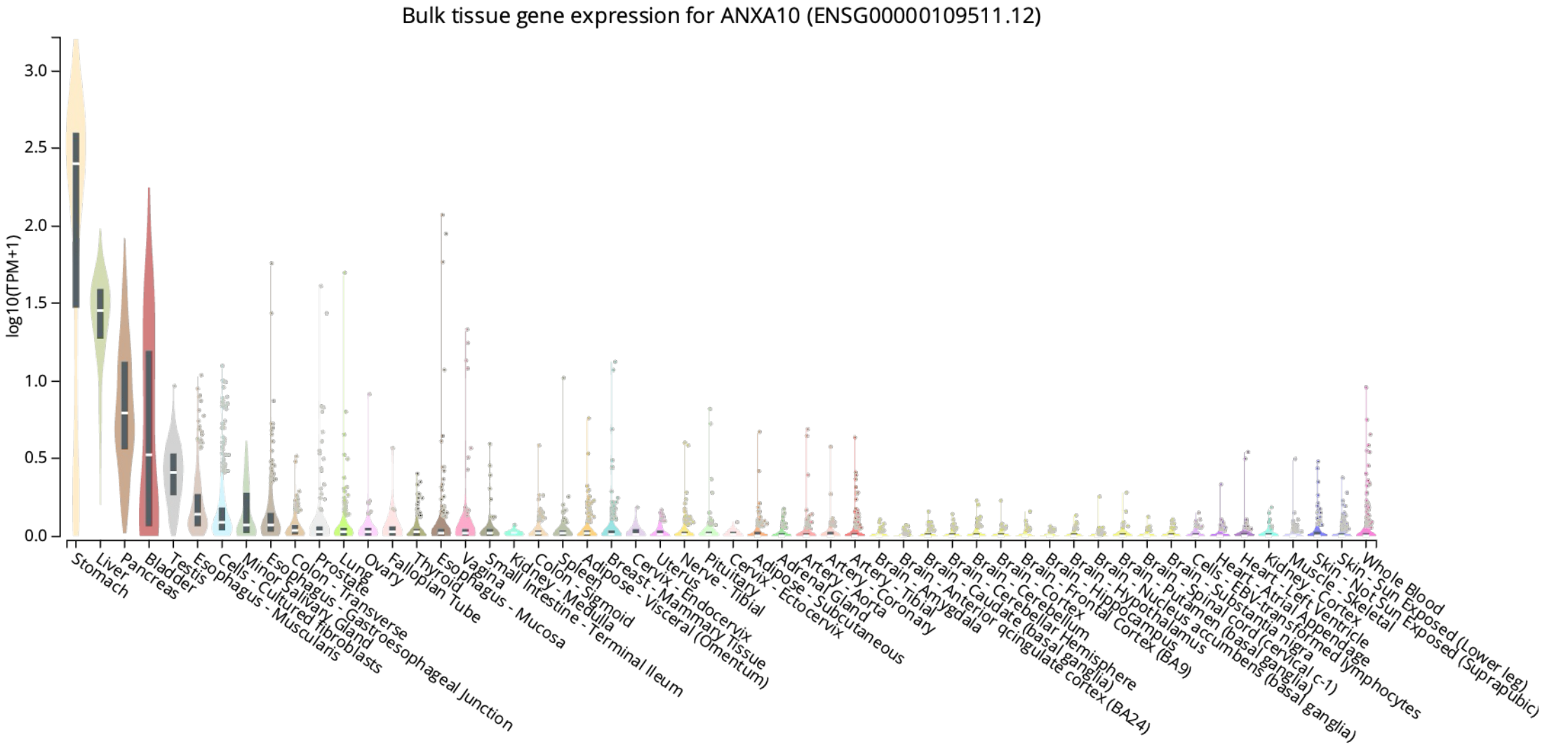

b

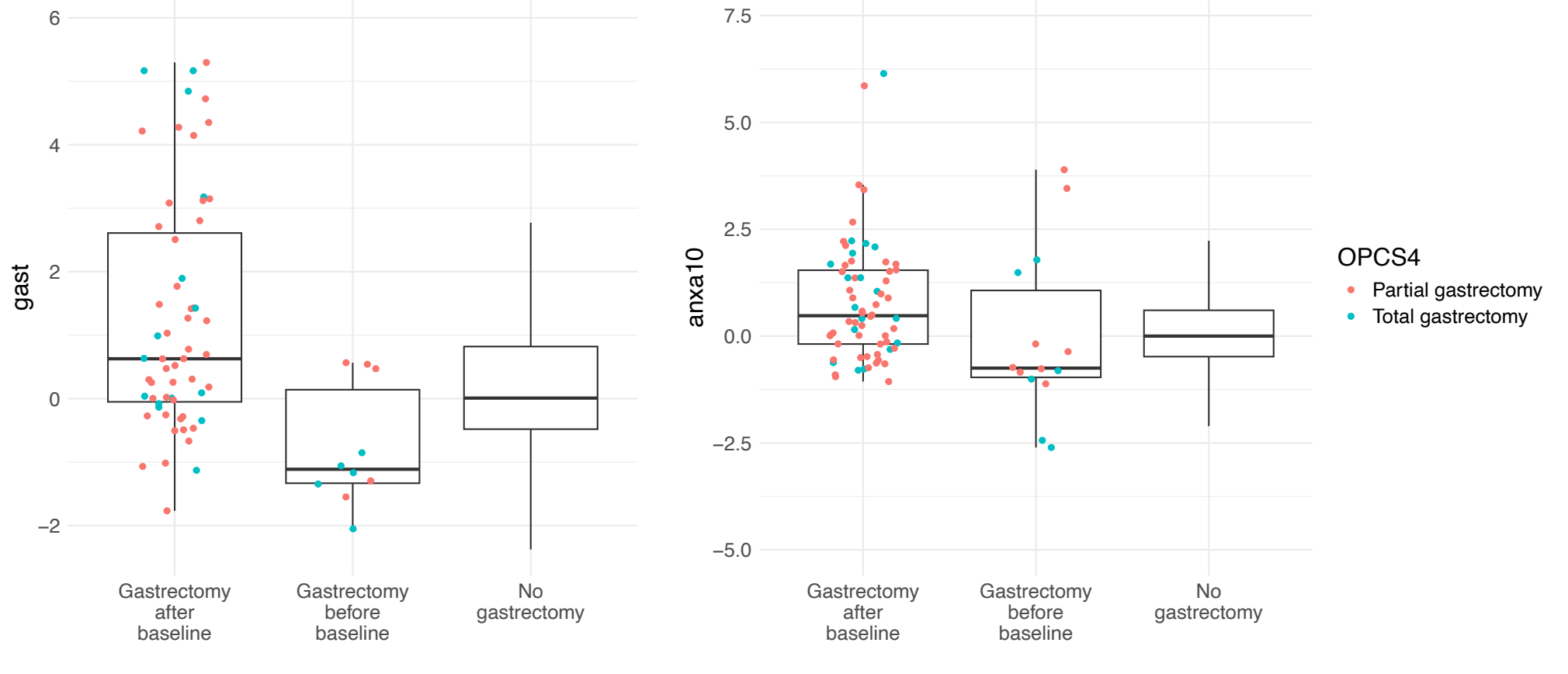

(a) Tissue-level gene expression plot for *ANXA10* from GTEx. The y-axis is in log scale. (Data Source: GTEx Analysis Release V10 (dbGaP Accession phs000424.v10.p2))

(b) Plasma levels of gastrin (left) and annexin A10 (right) in UK Biobank participants who had a gastrectomy before or after the baseline assessment, or no gastrectomy. Individual values for participants with gastrectomy also indicate whether it was a total or partial gastrectomy. Boxes show the median and IQR, and the whiskers extend to the furthest point within 1.5×IQR.

ESM Figure 8: Cell type origins of plasma ANXA10

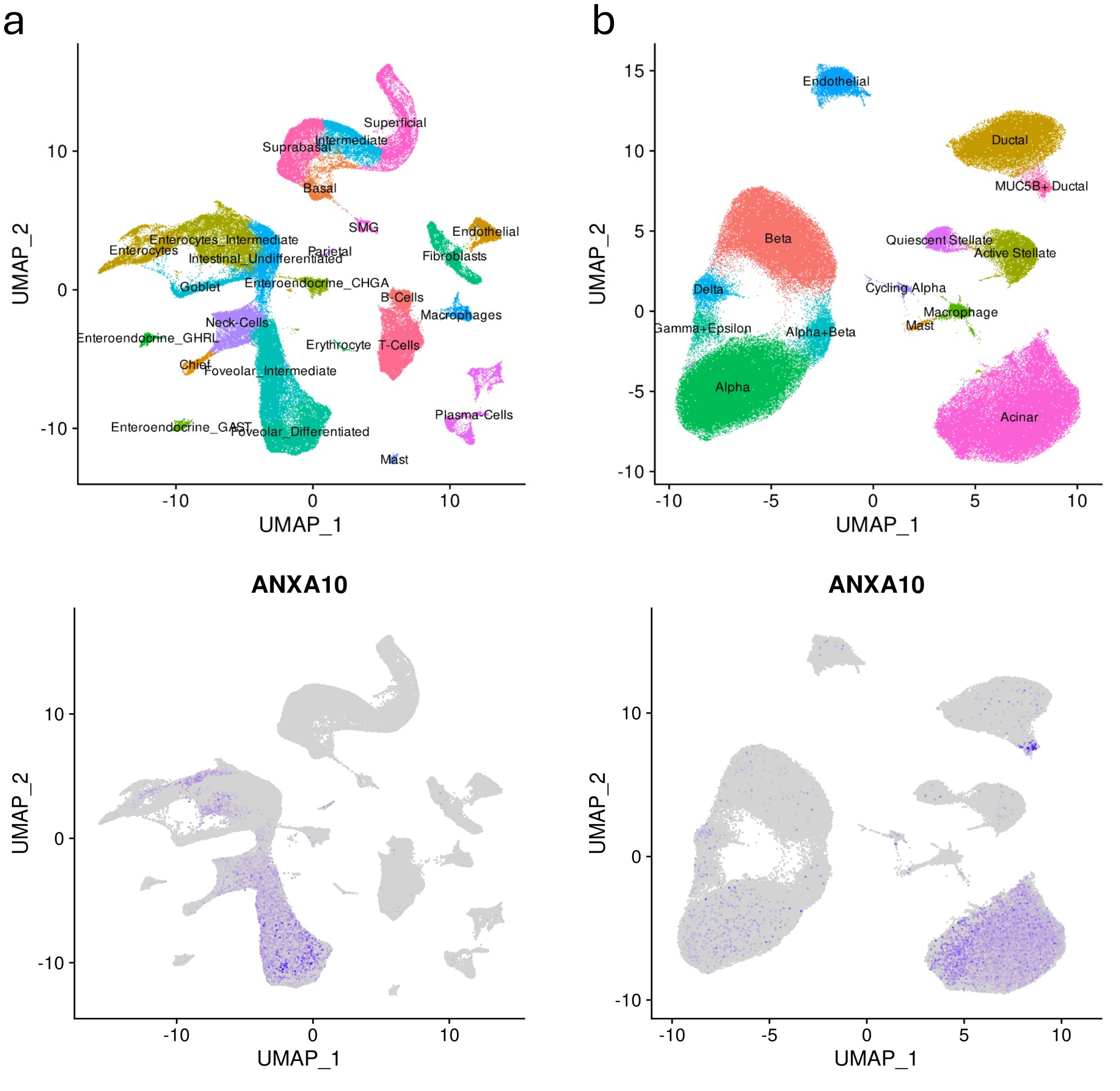

UMAP showing cell types (top) and feature plot of *ANXA10* expression (bottom), generated using public single cell RNA-seq datasets: (a) Gastrointestinal single cell data from Nowicki-Osuch et al. (b) Pancreas single cell data from Elgamal et al.

ESM Figure 9: Cell type origins of plasma ANXA10

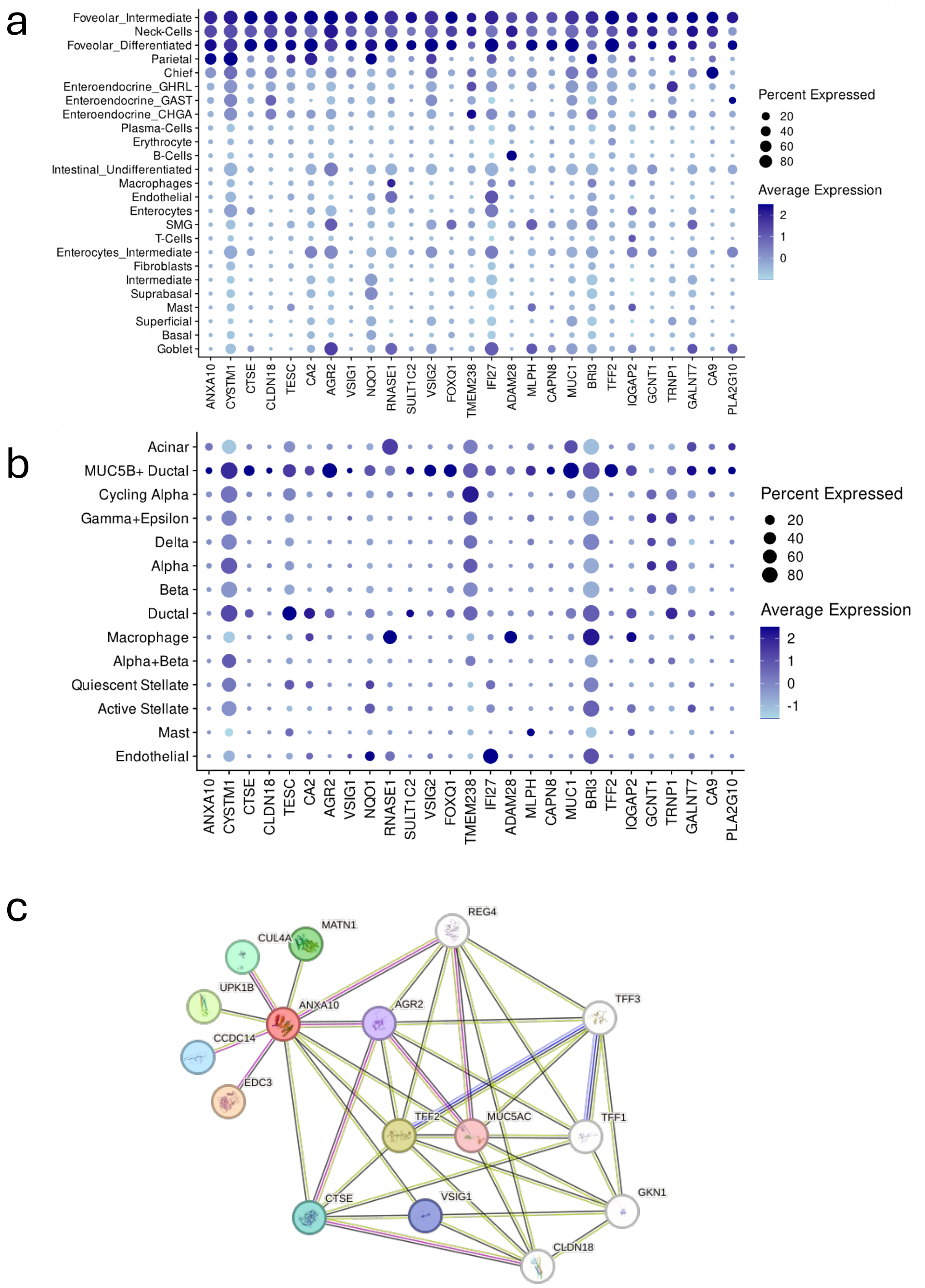

(a) Dotplot showing expression of the top 25 correlated genes with ANXA10 in healthy subset of gastrointestinal scRNA dataset (Nowicki-Osuch et al.).

(b) Dotplot of the same genes as in a, but in the non-diabetic subset of the pancreas scRNA dataset (Elgamal et al.). In a and b, CA9 and PLA2G10 were additionally plotted since they are in the top 95 percentile of all correlations in both datasets and also respond in the OGTT.

(c) STRING network of ANXA10 in human, with the settings: No more than 10 interactors in the first shell, and no more than 5 interactors in the second shell.

ESM Figure 10: Determinants of variance in ANXA10 and SCT plasma levels

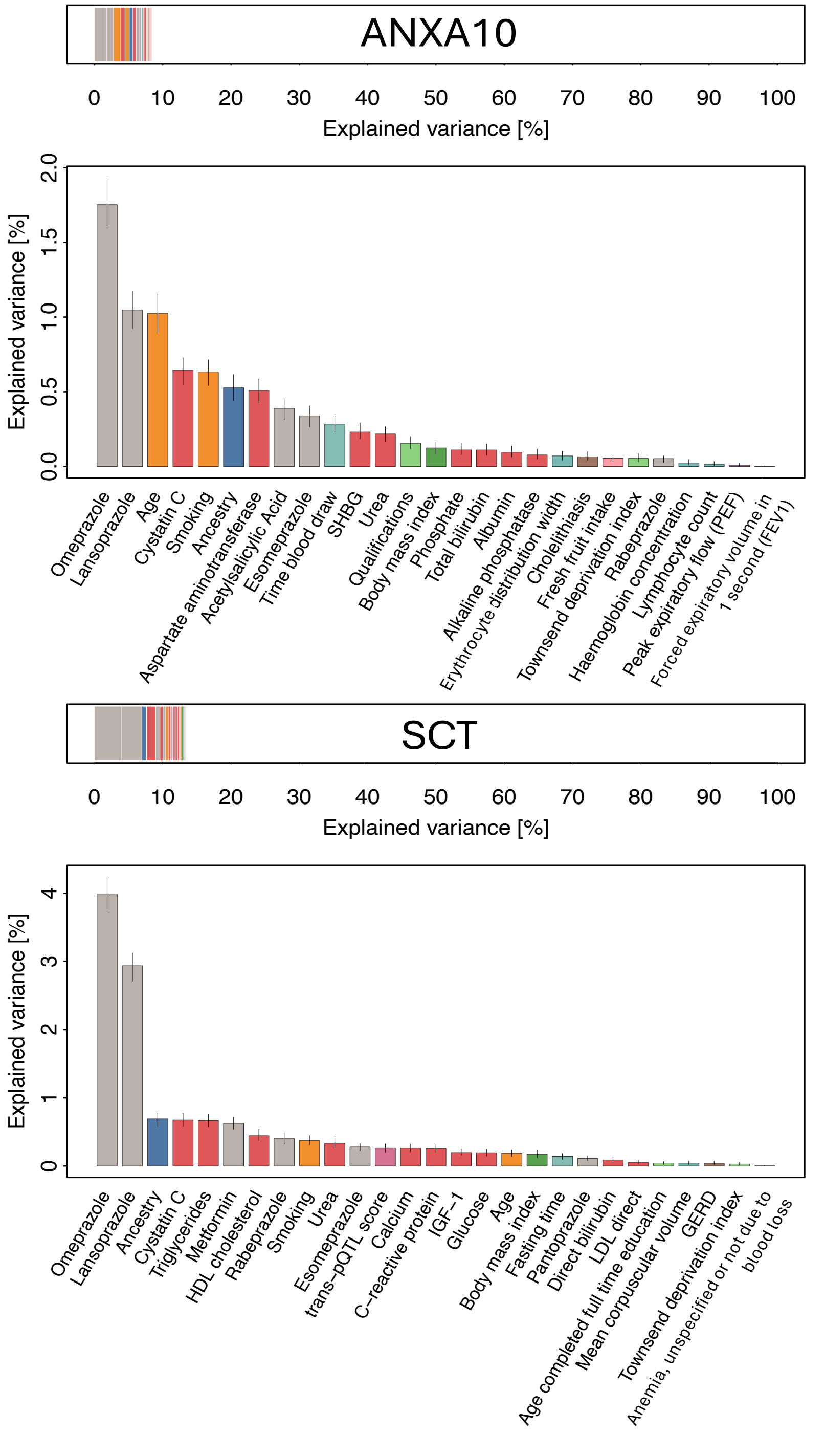

Percentage of plasma protein level variance explained by participant characteristics in UK Biobank based on R package ‘prodente’ from Pietzner et al. (2025), for ANXA10 (top) and secretin (bottom).

ESM Figure 11: Phenotypic associations with WARS pQTL

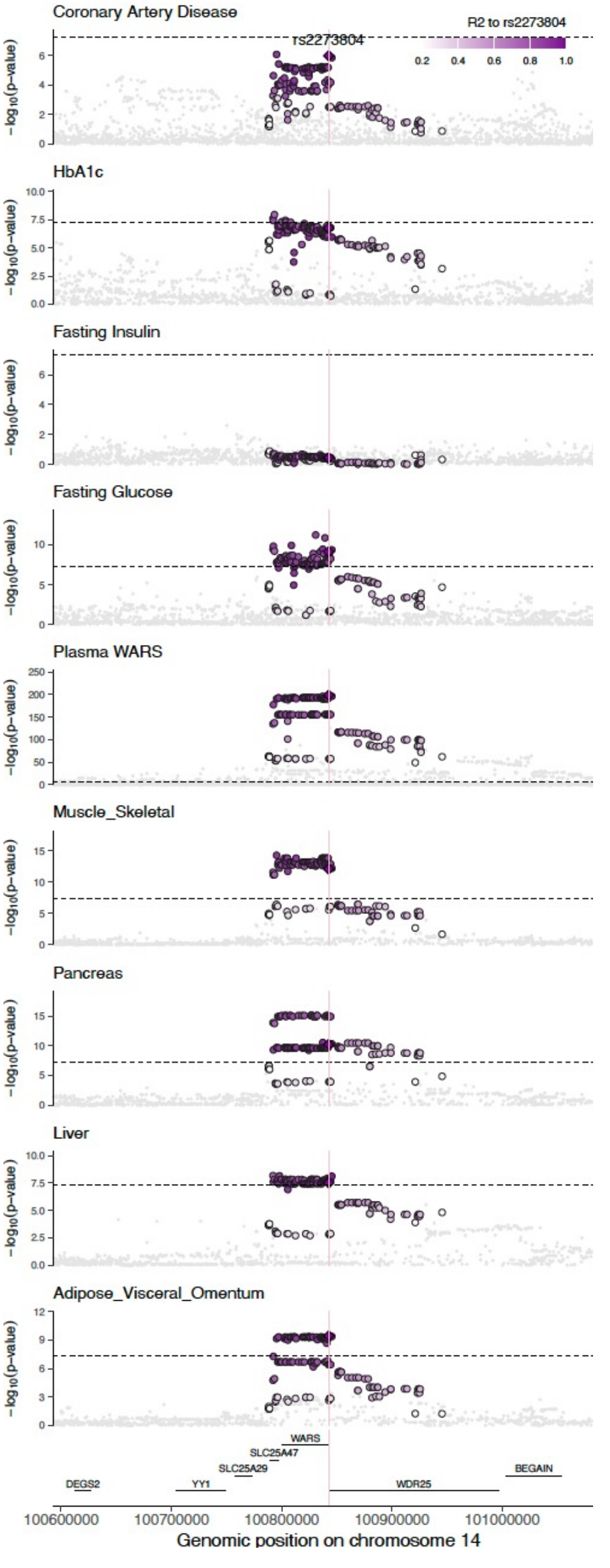

Regional association plots of each phenotype or molecular trait from Fig. 6b, displaying the 1 Mb region around rs2273804.
